# Co-Delivery of Eugenol and Dacarbazine by Hyaluronic Acid-Coated Liposomes for Targeted Inhibition of Survivin in Treatment of Resistant Metastatic Melanoma

**DOI:** 10.3390/pharmaceutics11040163

**Published:** 2019-04-03

**Authors:** Harshita Mishra, Pawan Kumar Mishra, Zeenat Iqbal, Manu Jaggi, Alka Madaan, Kimi Bhuyan, Namita Gupta, Neha Gupta, Karnika Vats, Ritu Verma, Sushama Talegaonkar

**Affiliations:** 1 Departmant of Pharmaceutics, School of Pharmaceutical Education and Research, Jamia Hamdard, New Delhi 110062, India; harshitasharma1088@gmail.com (H.M.); zeenatiqbal@jamiahamdard.ac.in (Z.I.); 2 Department of Wood Processing, Mendel University in Brno, 61300 Brno, Czech Republic; pawan.mishra@mendelu.cz; 3 Dabur Research Foundation, Ghaziabad 201010, India; manu.jaggi@daburresearch.in (M.J.); alka.madaan@daburresearch.in (A.M.); kimi.bhuyan@daburresearch.in (K.B.); namita.gupta@daburresearch.in (N.G.); neha.gupta@daburresearch.in (N.G.); karnika@daburresearch.in (K.V.); ritu.verma@daburresearch.in (R.V.); 4 Department of Pharmaceutics, Delhi Pharmaceutical Sciences and Research University, Govt. of NCT of Delhi, Pushp Vihar, New Delhi 110017, India

**Keywords:** Quality by Design (QbD), liposomes, hyaluronic acid, melanoma treatment, survivin inhibition, cytotoxicity, apoptosis, migration inhibition

## Abstract

While melanoma remains a challenge for oncologists, possibilities are being continuously explored to fight resistant metastatic melanoma more effectively. Eugenol is reported to inhibit survivin protein in breast cancer cells. Survivin is also overexpressed by melanoma cells, and is known to impart resistance to them against chemotherapy-induced apoptosis. To be able to fight resistant melanoma, we formulated hyaluronic acid (HA)-coated liposomes loaded with an effective combination of anti-melanoma agents (Dacarbazine and Eugenol), using a solvent injection method. Quality-by-Design (QbD) was applied to optimize and obtain a final formulation with the desired quality attributes, and within an acceptable size range. The optimized formulation was then subjected to performance analysis in cell lines. Coated-Dacarbazine Eugenol Liposomes were found to possess 95.08% cytotoxicity at a dacarbazine concentration of 0.5 µg/mL, while Dacarbazine Solution showed only 10.20% cytotoxicity at the same concentration. The number of late apoptotic cells was also found to be much higher (45.16% vs. 8.43%). Furthermore, migration assay and proliferation study also revealed significantly higher inhibition of cell migration and proliferation by Coated-Dacarbazine Eugenol Liposomes, signifying its potential against metastasis. Thus, surface-functionalized dacarbazine- and eugenol-loaded liposomes hold great promise against resistant and aggressive metastatic melanoma, with much less unwanted cytotoxicity and reduced doses of the chemotherapeutic agent.

## 1. Introduction

Melanocytes, while, on the one hand, protecting the skin from harmful ultraviolet radiation in their normal state, form one of the deadliest cancers when undergoing malignant growth on the other. Melanoma, which is the cancer of melanocytes, is a highly aggressive cancer, and causes up to 60–80% of skin cancer-related deaths [1,2]. As another matter of concern, the incidence rates of melanoma are continuously on the rise, with an increase of around 56% from 2005 to 2015 [3]. Furthermore, the median survival of metastatic melanoma (stage IV) patients is very poor, with no more than 10% of patients still being alive ten years after the treatment [4].

The major challenge associated with melanoma treatment is the resistance of melanoma cells chemotherapy, which can lead to the failure of the treatment, along with poor response and survival rates [5,6]. While several studies have been performed on the multidrug resistance of cancer cells [7,8], the inherent resistance of melanoma cells is reported to be due to a protein named survivin. The survivin protein is a member of the inhibitor of apoptosis (IAP) family, and exerts its effects by directly inhibiting caspases [9,10]. Several cancer types, including melanoma, have been reported to overexpress survivin. Survivin protects cancer cells from apoptosis, which is supposed to be induced by chemotherapy in order to kill cancer cells. Survivin is also known to play an essential role in angiogenesis by promoting the expression of the vascular endothelial growth factor (VEGF) in cancer cells [11]. It is reported that inhibiting the function of survivin in melanoma cells can spontaneously cause apoptosis, impairing the growth of the tumor [12]. Downregulation of survivin has also been found to inhibit migration, metastasis, and proliferation of cancer cells, both in vitro and in vivo [13]. The fact that it is overexpressed in most cancer cells, but hardly expressed at all in any normal tissue, makes it an attractive target for targeted anti-cancer therapies [14].

Eugenol (4-allyl-1-hydroxy-2-methoxybenzene), the main constituent of clove (*Syzygium aromaticum*), has been shown to target and inhibit survivin in breast cancer cells, thus inducing apoptosis and tumor inhibition [15]. Additionally, eugenol has specifically shown anti-proliferative and apoptosis-inducing effects in melanoma in vitro and in vivo [16]. The presence of a hydroxyl group and an aromatic ring in the eugenol structure has been reported to be important for its anti-cancer action [17].

Nanotechnology has a special role in the treatment of cancer, because it enables scientists to specifically target the cancer cells with anti-cancer drugs, sparing normal tissues, and avoiding any unwanted side effects, which constitute the major drawback of chemotherapy [18,19]. Nanoagents loaded with chemotherapeutic drugs and surface-functionalized with ligands, have been formulated and successfully implemented in the treatment of melanoma [19,20,21,22]. However, the design and optimization of a formulation in the most effort-, time- and cost-effective manner have proven challenging for scientists. Here, the QbD (Quality by Design) approach comes to the rescue. QbD is a scientific and systematic approach for use in the development of pharmaceutical formulations. It involves defining and taking into consideration all of the parameters that critically affect the final quality and performance of the formulation. QbD can be focused on defining the aspects of quality that need to be optimized [23]. Application of QbD helps in understanding and establishing the relationship between the process parameters and the quality attributes of the formulation. It helps scientists understand how the critical process parameters should be varied in order to consistently produce a pharmaceutical formulation with the desired quality attributes [23,24].

Bearing the above-stated facts in mind, we formulated dual-loaded, surface-functionalized liposomes for the targeted anti-resistance therapy of melanoma. Dacarbazine, an alkylating chemotherapeutic agent, is still the mainstay of melanoma treatment, and it forms the basis for most anti-melanoma combinations [4]. Dacarbazine is combined with eugenol for its survivin-targeting ability. Both drugs are loaded in liposomes, and liposomes are finally surface-functionalized with Hyaluronic acid (HA) in order to enable the active targeting of CD44 receptors, which are overexpressed by cancer cells [25].

In this case, since two drugs were to be loaded into the liposomes, the QbD was applied on two levels. On the first level, single drug (dacarbazine)-loaded liposomes were optimized, and on the second level, dual-loaded (Dacarbazine + Eugenol) liposomes were optimized by fixing several parameters based on the results of the first-level QbD. Applying QbD on two levels, and optimizing all the independent variables in a two-step QbD approach, rather than one, made the whole process more cost-effective and more reproducible.

## 2. Materials and Methods

*Chemicals*: Dacarbazine was kindly provided by Intas Pharmaceuticals (Ahmedabad, India). Eugenol (99.9%) was purchased from Sigma Aldrich (St Louis, MO, USA). Lipoid S100 was a generous gift from Lipoid GmbH, Frigenstr. 4, 67065 Ludwigshafen, Germany. All other reagents used were of analytical grade and were used without any further purification.

*Cell Lines*: Cell line studies were carried out in the Cell Biology Department, Dabur Research Foundation, Ghaziabad, India. SK-MEL-28 and B16F10 melanoma cell lines were procured from the National Center for Cell Science (NCCS, Pune, India); The EA.hy.926 cell line was procured from the American Type Culture Collection (ATCC, Rockville, MD, USA).

### 2.1. Synthesis of Liposomes

**Figure 1:**
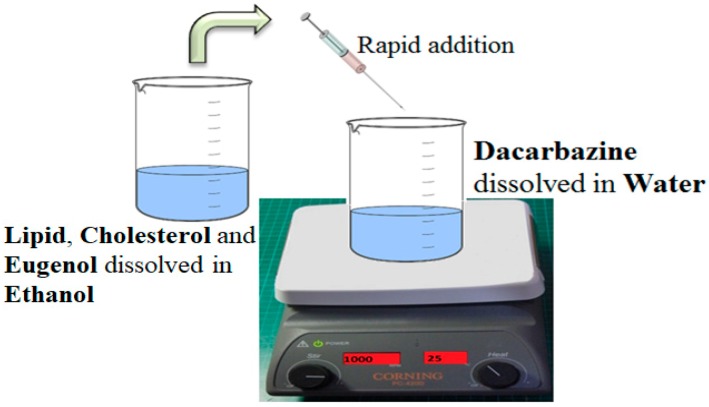
Method of Preparation of dual loaded Liposomes. An ethanolic solution of lipid, cholesterol and the lipophilic drug is rapidly injected into an aqueous solution of a hydrophilic drug, under stirring. Spontaneous formation of liposomes takes place.

Liposomes were synthesized by a solvent injection method using ethanol as the organic solvent [26]. Lipid and cholesterol were dissolved in ethanol. This constituted the organic phase. Then the eugenol was dissolved in this organic phase because of its lipophilic nature. Separately, dacarbazine was dissolved in distilled water, which constituted the aqueous phase. This aqueous phase was then kept on stirring (1000 rpm), and the organic phase was rapidly injected into it using a syringe of 1 mL capacity, and a 24 gauge needle size. The volume of ethanol was fixed at 5 mL. Figure 1 gives a pictorial representation of the method of preparation of dual drugs-loaded liposomes.

### 2.2. Optimization of Formulation: Quality-by-Design (QbD) 

To synthesize the dual loaded liposomes with optimum parameters, Central Composite Design (CCD) was chosen and applied using Design Expert^®^ version 11.0.0 by Stat-Ease, Inc. (Suite 480, Minneapolis, MN, USA). (CCD) was selected for the optimization of the formulation because it generates a greater number of runs as compared to other designs in the Design Expert software [27]. Since the formulation had to be loaded with two drugs and specific characteristics were desired, the central composite design was applied at two levels. 

Each variable was set at low (−1) and high (1) levels. The software itself took a middle value (0) also, and generated combinations with three different values, −1 (low), 0 (medium) and +1 (high).

#### 2.2.1. First Level

##### Initial Risk Assessment

Critical quality attributes (CQAs) are the quality aspects of the final product which are critical for its performance, and are to be optimized. In the present study, particle size and entrapment efficiency were selected as CQAs for initial risk assessment. Critical material attributes (CMAs) and critical process parameters (CPPs) are the material and process variables, respectively, that are expected to affect and alter the quality (CQAs) of the final formulation.

According to the literature surveyed and results of the preliminary experiments, various CMAs and CPPs were identified, namely:

CMAs: Lipid concentration, Drug concentration, Lipid: Cholesterol
CPPs: Water: Ethanol, Stirring speed, Stirring time

The effect of all these CMAs and CPPs was assessed to identify the intensity of their impact, and the appropriate range of each variable. The conclusions are summarized in Table 1.

##### Design of the Experiment

The first level design of experiment (DOE) was applied first to optimize single drug (dacarbazine)-loaded liposomes to select the optimum lipid concentration and waterethanol ratio to synthesize liposomes with minimum size and good entrapment of the base drug, i.e., dacarbazine.

As stated in Table 2, three independent variables (denoted as Factors) were selected. Factor 1 was Lipid concentration (mg/mL), i.e., amount of lipid to be taken and dissolved in ethanol. Low level (−1) was fixed at 10 mg/mL, and high level (+1) was fixed at 30 mg/mL. Factor 2 was the water:ethanol ratio, with a low-level value of 3 (−1), and a high-level value of 5 (+1). Factor 3 was the drug concentration, i.e., the amount of dacarbazine that needs to be dissolved in water. The values selected for this drug concentration were 1 mg/mL (low level, −1) and 3 mg/mL (high level, +1). Two dependent variables (final characteristics of the formulation, denoted as Responses) were selected, which were the main criteria for the suitability of the formulations. Response 1 was size, and response 2 was entrapment efficiency of the dacarbazine.

The lipid:cholesterol ratio, stirring speed and stirring time were taken as fixed variables. By preliminary analysis of factors affecting the formulation outcomes, stirring time and stirring speed were fixed at 60 min and 1000 rpm respectively, as it was found that these values produced optimum results. The lipid:cholesterol ratio was also fixed at 2, in accordance with the results obtained in the preliminary studies.

#### 2.2.2. Second Level

##### Initial Risk Assessment

In addition to the variables fixed previously, lipid concentration and water:ethanol ratio were also fixed in the second level CCD. The values of these two factors were fixed at the optimum values as suggested by the first level CCD. All values are summarized in Table 3.

##### Design of Experiment

Second level DOE was applied to determine the right concentration of both of the drugs to be taken in order to produce liposomes with the minimum size and a maximum ratio of entrapped eugenol to entrapped dacarbazine (Eugenol:Dacarbazine). Two independent variables (factors) were selected; Factor 1 was a concentration of dacarbazine, and Factor 2 was the concentration of eugenol. The fixed values of Factor 1 and Factor 2 are shown in Table 3.

Three-dimensional response surface plots were obtained using the software to illustrate the effect of selected factors (independent variables) on the responses (dependent variables). Analysis of Variance (ANOVA) was applied to the obtained responses. The equations for each independent variable were generated by using the values of its coefficients.

The values of responses obtained were fitted into different models, namely, linear, two-factor interaction (2F1), quadratic and cubic models. Based on the data obtained from the lack of fit tests and model summary statistics, a suitable model was selected and applied. Constraints were applied on dependent variables, and optimized formulation with the highest desirability factor was selected using the numerical technique.

### 2.3. Surface Functionalization

To actively target the liposomes to cancer cells, the surface of optimized dual loaded liposomes was coated with hyaluronic acid (HA), which has a particular affinity for CD44 receptors that are overexpressed by most of the cancer cell lines. HA is anionic due to the presence of carboxyl groups, and to employ the ionic interaction method for coating, the liposomes had to have a cationic surface. The liposomes prepared using Lipoid S100 were anionic, and had a negative surface charge as revealed by zeta potential studies. So, to make cationic liposomes, CTAB (cetyl tetra ammonium bromide) was used. Briefly, 10 mg of CTAB was dissolved along with lipid, cholesterol, and eugenol in the ethanol. This ethanolic phase was added to an aqueous phase containing dacarbazine under stirring. The ethanol was later evaporated to obtain drugs-loaded cationic liposomes.

Separately, HA solutions of four different concentrations (0.005%, 0.01%, 0.05%, 0.1%) were prepared by dissolving certain quantities of HA in water, and stirring for 60 min. To coat the HA on liposomes, 10 mL of optimized cationic liposomal suspension was added into 5 mL of the HA solution. The addition was done under stirring, and stirring was continued for four hours [28].

### 2.4. Particle Size, Size Distribution, and Zeta Potential

Blank liposomes, dual loaded liposomes, and dual loaded surface-coated liposomes were scanned for the said parameters. The mean particle size, poly dispersity index (PDI) and zeta potential of the different liposomes were determined by dynamic light scattering using a particle size analyzer (Delsa™ Nano C, Beckman Coulter Counter, Brea, CA, USA). The liposomal suspension was diluted ten times using deionized water, and this diluted suspension was put into the particle size analyzer to obtain the results [29].

### 2.5. Electron Microscopy

To confirm the size of the liposomes and ascertain the successful coating on the surface, electron microscopic analysis was performed. For Scanning Electron Microscopy (SEM), the sample was coated with gold, and then kept in the sampling unit as a thin film. The photographs were taken at different magnifications using a Scanning Electron Microscope (Jeol, Tokyo, Japan) [30]. For Transmission Electron Microscopy (TEM), a drop of this sample was deposited onto a copper grid coated with fomvar. The grid was then immersed in one drop of 2% phosphotungstic acid for 20 s and then was allowed to dry. The grid was finally observed under Transmission Electron Microscopy (Tecnai, G20, FEI, Eindoven, The Netherlands).

### 2.6. Drug Loading and Entrapment Efficiency

To determine the loading of drugs in the synthesized liposomes, the liposomal suspension was centrifuged at 36,000 rpm (Beckman Coulter, Optima™ L-100K, London, United Kingdom) to remove the unentrapped drugs. The supernatant which contained unentrapped drugs was separated, and the pellets of liposomes were dissolved in ethanol. Ethanol, which could dissolve the lipid as well as both drugs, was a suitable solvent for the determination of drug loading. Since a simultaneous loading of two drugs was to be determined, a novel UV absorptivity method (Shimadzu, Kyoto, Japan) for simultaneous determination of the drugs, was developed by the authors. The ethanolic solution of drugs-loaded liposomes was suitably diluted, and its absorbance was measured at λ_1_^et^ (λ_max_ of dacarbazine in ethanol, i.e., 333 nm) and λ_2_^et^ (λ_max_ of eugenol in ethanol, i.e., 282.5 nm) against an ethanolic solution of unloaded liposomes as blank. These absorbance values (A_1_^et^ and A_2_^et^) were put into Equations (1) and (2) which were generated by the absorptivity method.

A_1_^et^ = 1005 C_d_^et^ + 72.24 C_e_^et^ (1)

A_2_^et^ = 616 C_d_^et^ + 144.21 C_e_^et^ (2)

After solving the simultaneous equations, amount of dacarbazine and amount of eugenol present in the formulation were determined by multiplying the concentration of dacarbazine (C_d_^et^) and the concentration of eugenol (C_e_^et^) with dilution factors.

Drug loading of the formulation with respect to both drugs was calculated by using the following formula:

Drug Loading (%) = (Amount of drug present in formulation/Total weight of the formulation × 100)

After determining the amount of drugs present in the liposomes, the entrapment efficiency of both drugs was calculated by the following formula:

Entrapment efficiency (%) = (Amount of drug entrapped/Total amount of drug used) × 100

### 2.7. In Vitro Drug Release

The in vitro release study of dual loaded surface-functionalized liposomes was carried out throughout 72 h, using the dialysis bag method [31]. Phosphate Buffer Saline (PBS) (pH 7.4): Propylene glycol (9:1) was used as a release medium because both the drugs were soluble in this media, while the lipid was insoluble. The dialysis membrane (MW cut off 8–10 kDa; Spectra/Por^®^ Spectrum Laboratories, Inc., Visalia, CA, USA) was activated before using as per the instructions given on the packaging. The liposomal suspension was centrifuged as described above. The supernatant was discarded, and pellets were dispersed in 10 mL of release media. This dispersion was put in a dialysis bag, and the bag was suspended in 200 mL of receiving phase i.e., PBS (pH 7.4): Propylene glycol (9:1), and placed into an incubator shaker maintained at 37 °C and 100 rpm. Aliquots each of 3 mL were withdrawn at various time points (up to 72 h). The same volume (3 mL) of the media was replaced after each sampling to maintain the sink condition during the study. The absorbance of samples withdrawn at different time points (and suitably diluted when needed), was measured at λ_1_ (λ_max_ of dacarbazine in release media, i.e., 331 nm) and λ_2_ (λ_max_ of eugenol in release media, i.e., 281.5 nm) against pure release media as blank. These absorbance values (A_1_ and A_2_) were put into Equations (3) and (4), which were generated by an absorptivity method developed in house using PBS: Propylene glycol (9:1) as a solvent.

A_1_ = 949.64 C_d_ + 30.59 C_e_ (3)

A_2_ = 319.93 C_d_ + 138.36 C_e_ (4)

After solving the above simultaneous equations, the amount of both the drugs (C_d_ and C_e_) present in the release media at different time points was calculated.

% Release at any point of time = (Amount of drug present in the release media/Total amount of drug present in formulation/dialysis bag) × 100

### 2.8. Stability Study

To check the stability of the final formulation, the liposomes were lyophilized and stored under refrigeration (4 °C) for four weeks. The particle size, PDI and drug content of the liposomes were determined at the end of every week by dispersing in PBS (7.4) to assess the storage stability of the liposomes. Stability of the liposomes was also assessed in cell culture media (Eagle’s minimal essential medium (DMEM) + 10% FBS) for three days.

### 2.9. Cell Line Studies

Growth medium used for cell lines was DMEM + 10% FBS. Growth conditions were 37 °C, 95% Humidity, 5% CO_2_. Sub-culturing was done once the cells were 80–90% confluent in the T-75 culture flask. Untreated cells with complete medium (10% FBS) served as our complete medium control, cells with sera free medium (SFM) served as an SFM control, and the cells treated with Paclitaxel/Doxorubicin served as the Positive control.

#### 2.9.1. MTT Assay

The MTT assay was first performed on the SK-MEL-28 melanoma cell line using the previously reported method [31]. The four samples tested were blank liposomes (BL), Dacarbazine solution (DS), Dacarbazine Liposomes (DL), and Dacarbazine + Eugenol Liposomes (DEL). To perform the assay on above-said test samples, cells were plated at a density of 0.5 million/well in 6-well culture plates, and were incubated for 24 h in a CO_2_ incubator. 20 μL of 5 mg/mL of MTT 3-(4,5-dimethythiazol-2-yl)-2,5-diphenyl tetrazolium bromide solution was added to all the wells, followed by additional incubation for three h at 37 °C. The supernatant was aspirated, and 150 μL of dimethyl sulfoxide (DMSO) was added to each well to dissolve the formazan crystals. The absorbance of each well was then read at 540 nm using a Synergy HT micro plate-reader.

The percentage cytotoxicity corresponding to each treatment was calculated using the following formula:

% Cytotoxicity = [*R* − *X*)/*R*] × 100

where *X* = Absorbance of wells corresponding to treated cells; *R* = Absorbance of untreated cells (cells maintained in DMEM + 10% FBS).

Furthermore, in addition to the above said formulations, the cytotoxicity of final coated Dacarbazine + Eugenol Liposomes (DELC) was assessed in B16F10 melanoma cells using the same procedure as described.

#### 2.9.2. Apoptosis Assay

Apoptosis profile of the SK-MEL-28 cells treated with formulations was studied by flow cytometry using Annexin V. Growth medium and growth conditions were kept the same as before. Cells were plated at a density of 0.5 million/well in 6-well culture plates, and were incubated for 24 h in a CO_2_ incubator. Cells were sera starved in DMEM + 0%FBS for four hours, and were then treated with test samples (DS, DL, DEL, Coated Dacarbazine Liposomes (DLC), and DELC) for 24 h. After 24 h, cells were processed for Annexin V assay as follows: Cells were harvested very gently by trypsinization and centrifuged at 300 *g* for 5–7 min. The cell pellet was resuspended in 200–300 µL of Phosphate Buffer Saline (PBS). 100 µL of cell suspension was transferred into pre-labeled tubes for staining. 100 µL of Nexin reagent (Annexin V/7-AAD, Guava technologies, Merck millipore, Danvers, MA, USA) was added to each tube and mixed gently. Samples were incubated for 20 min at room temperature in the dark. Samples were then acquired on a flow cytometer (Guava Technologies, Hayward, California, USA).

#### 2.9.3. Migration Assay

Cell migration analysis was done by the wound healing method on Ea.Hy293 cells. EA.hy926 cells were counted using a hemocytometer and plated in 12-well plates at the density of 0.2 × 10^6^ cells/well in medium + 10% FBS. The cells were incubated overnight in the CO_2_ incubator to allow cell recovery and exponential growth. Following overnight incubation, these cells were sera starved (DMEM + 0%FBS) for six h. After six h, the cells were washed with PBS, and a small linear scratch (representative wound) was created in the confluent monolayer (middle of the well) by gently scraping with sterile 200 μL micropipette tip. Photomicrographs of the scratch were taken at 0 h (Initial time point). Cells were rinsed with serum-free DMEM and grouped for treatment with test samples (DS, DL, DEL, DLC, DELC). Photomicrographs of the scratch were taken at 0 h, 24 h, 48 h, and 72 h. 

The photomicrographs obtained were analyzed for quantitative assessment of the area of wound closure using ImageJ tool software. Percentage migration with respect to untreated cells at different time points was calculated using the following formula:

% Migration = [(MigrationUntreated − MigrationSample)/MigrationUntreated] × 100

The extent of inhibition in cell migration with respect to untreated cells at different time points was calculated using the following formula:

% Inhibition = 100% − % Migration of Untreated sample

#### 2.9.4. Proliferation Assay

A proliferation assay also was performed on EA.hy926 cells. After sub culturing, the cells were counted using the hemocytometer and plated in 96 well plates at the density of 1 × 10^5^ cells/well/180 μL of the growth medium with 10% FBS. After 24 h of incubation in a CO_2_ incubator, these cells were sera starved by replacing the medium with 0% FBS. The cells were incubated for 24 h. After 24 h, they were separately treated with 5 test items (DS, DL, DEL, DLC, DELC) at different concentrations in medium +10% FBS. After three days of incubation, the effect of test formulations on cell proliferation was determined by calculating the % viability of cells using MTT assay. Serum-free media control cells were assessed with respect to complete medium control cells.

### 2.10. Statistical Analysis

All the experiments were performed in triplicate. Excel™ program 2010 (Microsoft™, 36 Redmond, WA, USA) was used to calculate the mean ± standard deviation of the obtained data. Data are presented as the mean ± standard deviation (SD). All statistical analyses were carried out with Statistica 13 (TIBCO Software Inc., Palo Alto, CA, USA), using one-way Analysis of Variance (ANOVA) and Tukey’s HSD as the posthoc test. All differences were considered significant at *p* < 0.05.

## 3. Results and Discussion

### 3.1. Synthesis

As mentioned earlier, the liposomes were synthesized by solvent injection method. As per the experiments conducted by [32], this solvent injection method produces smaller-sized liposomes with comparatively higher encapsulation efficiencies of the entrapped drug. Lasic [33] had explained the bilayer planar fragments (BPFs) theory for the mechanism of formation of liposomes by the ethanol injection method. According to this theory, the lipids which are dissolved in ethanol precipitate at the phase boundary of water and ethanol (organic solvent), resulting in the formation of BPFs. When the organic solvent is completely diffused in the external aqueous phase, vesicle formation takes place consequent to self-assembly of BPFs.

### 3.2. Optimization Using QbD

The formulation was optimized by following the QbD approach. QbD is a more economical and time effective method, and thus has replaced the hit and trial method for formulation optimization [27]. DOE (Design of Experiment) is an integral part of the QbD approach, which involves the use of the software to generate “structured” data tables. The software enables us to obtain a graphical interpretation of the results and effect of each parameter on the critical quality attributes (CQAs) of the formulation. Central composite design (CCD) was chosen because it generates better factorial design. Also, CCD can be used for working on factors as small as two in number.

#### 3.2.1. First Level

At the first level, only ardacarbazine-loaded liposomes were prepared and optimized in order to find out the optimum value of lipid concentration and water:ethanol ratio. CQAs selected were the size of the liposomes and the entrapment efficiency of the drug as both these are crucial parameters significantly influencing the performance of the formulation.

After putting minimum and maximum values of the independent variables into the CCD statistical experimental design, the software suggested 20 runs with five center points. These 20 formulations were prepared, analyzed, and the obtained values of dependent variables for each run were put into the table (Table 4). Software-generated polynomial equations, which could predict the effect of individual factors (independent variables), as well as combinations of factors on the responses (dependent variables).

##### Response Analysis for Optimization

Size of any nanoformulation is a crucial parameter, as it has major impacts on the performance, targeting ability and fate of the formulation in the body. Also, since finally, two drugs were to be loaded in the liposomes, and then coating was also to be done; the size of the liposomes had to be maintained at the minimum from the beginning. On fitting the resulted response data to various models, it was found that the data were best explained by the quadratic model. Summarized in Table 5, the Model *F*-value of 57.79 implies that the model is significant, indicating a considerably significant effect on dependent variables. *P*-values less than 0.0500 indicate that model terms are significant. The Lack of Fit *F*-value of 1.36 implies that the Lack of Fit is not significant relative to the pure error. Non-significant lack of fit is favorable. Value of Correlation Coefficient (*R*^2^) of 0.9811 suggested a good fit of the model as it approaches 1. Also, the Predicted *R*^2^ of 0.9380 and the adjusted *R*^2^ of 0.9642 are in reasonable agreement, i.e., the difference is less than 0.2.

Equation (5) represents the effect of independent variables on the size of the final liposomes.

Size = +81.07 + 17.60 × A − 4.40 × B + 2.34 × C − 0.2450 × AB − 0.0500 × AC + 0.2850 × BC + 11.59 × A^2^ + 0.4245 × B^2^ − 4.22 × C^2^ (5)

This equation in terms of coded factors can be used to make predictions about the response for given levels of each factor. The coded equation is useful for identifying the relative impact of the factors by comparing the factor coefficients.

**Figure 2:**
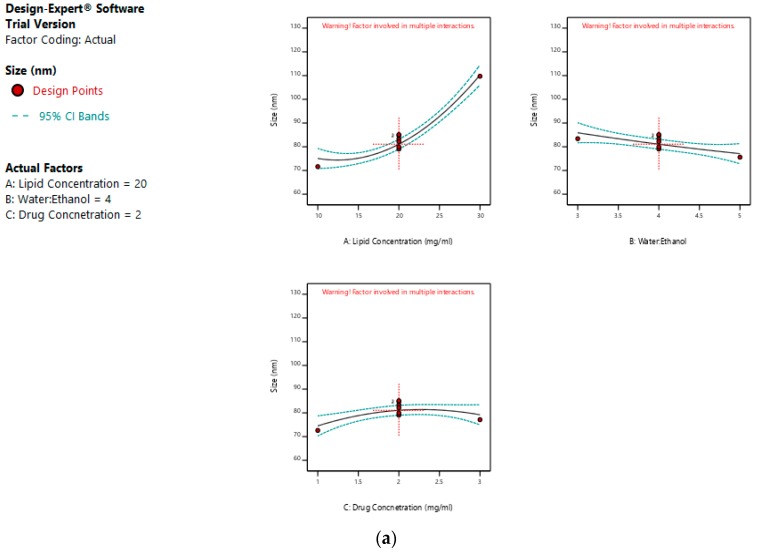

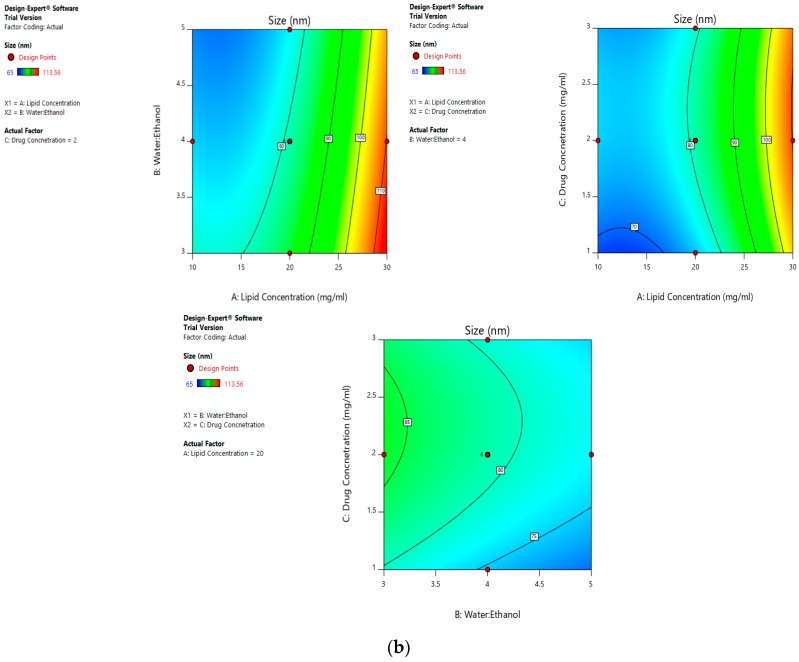

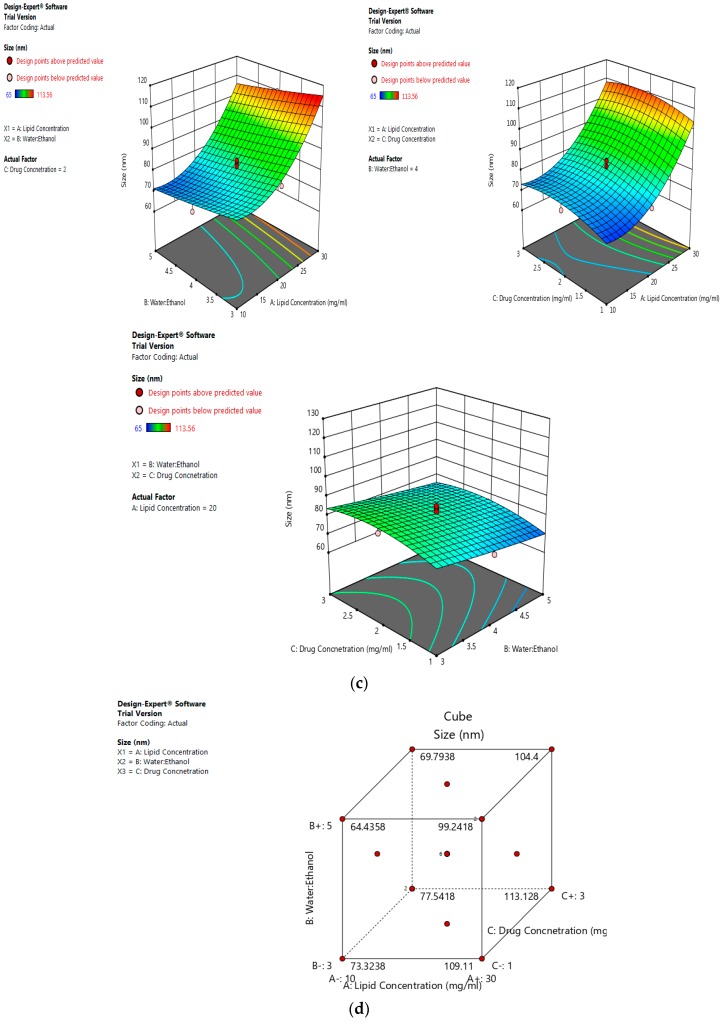
Effect of factors on the size of the dacarbazine liposomes (DL) (**a**) Effect of individual factors on Size; (**b**) Effect of combinations of two factors on Size (2D plots); (**c**) Effect of combinations of two factors on Size (3D plots); (**d**) Effect of all three factors on Size.

The mean size ranged from 65 nm to 113.56 nm. The values of the coefficients of A, B, and C in the equation above suggest that the size of the liposomes is most influenced by the lipid concentration, followed by its water:ethanol ratio, and least affected by the drug concentration. The linear terms of lipid concentration and drug concentration have a positive effect on size, whereas the linear term of the water:ethanol ratio has a negative effect. Thus, the size of the liposomes increases with an increase in lipid concentration and drug concentration, and decreases with any increase in its water:ethanol ratio. Figure 2a–d represents the effect of independent variables on the size of liposomes.

The entrapment efficiency of the drug should be constrained at maximum to make formulation and the whole process more cost-effective. On fitting the response data to various models, the data was best explained by the quadratic model (Table 5). Values of Model F-value, Lack of fit *F*-value and *p*-value are also favorable (Table 5).

Equation (6) represents the effect of independent variables on the entrapment efficiency of dacarbazine liposomes. 

Entrapment Efficiency = +25.55 + 3.13 × A − 0.8070 × B + 2.36 × C − 3.89 × AB − 1.41 × AC − 0.7075 × BC − 1.93 × A^2^ − 0.7691 × B^2^ − 0.7741 × C^2^ (6)

Values of entrapment efficiency ranged from 11.14% to 31.67%. Same as size, the linear terms of lipid concentration and drug concentration have a positive effect on entrapment efficiency, whereas the linear term of this water:ethanol ratio has a negative effect. As evidenced by the values of the coefficients, the entrapment efficiency is most effected by the lipid concentration, followed by drug concentration and the water:ethanol ratio. As the lipid concentration increases, numerous and larger liposomes are formed, thus higher is the entrapment efficiency. Also, when the drug concentration is increased, more drug is entrapped in the liposomes. 

**Figure 3:**
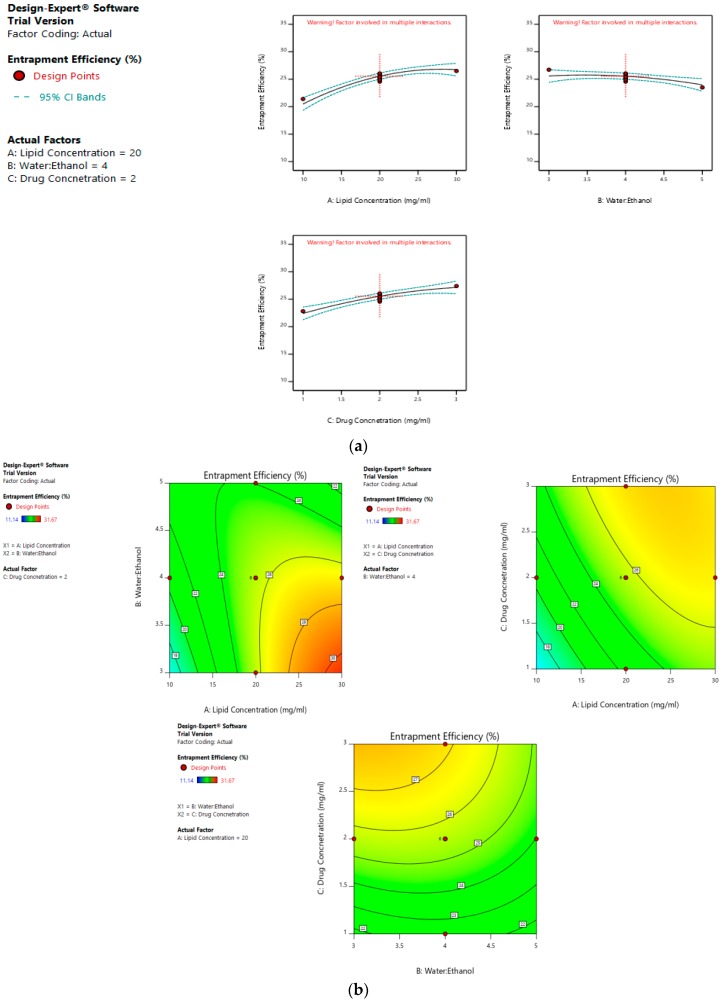

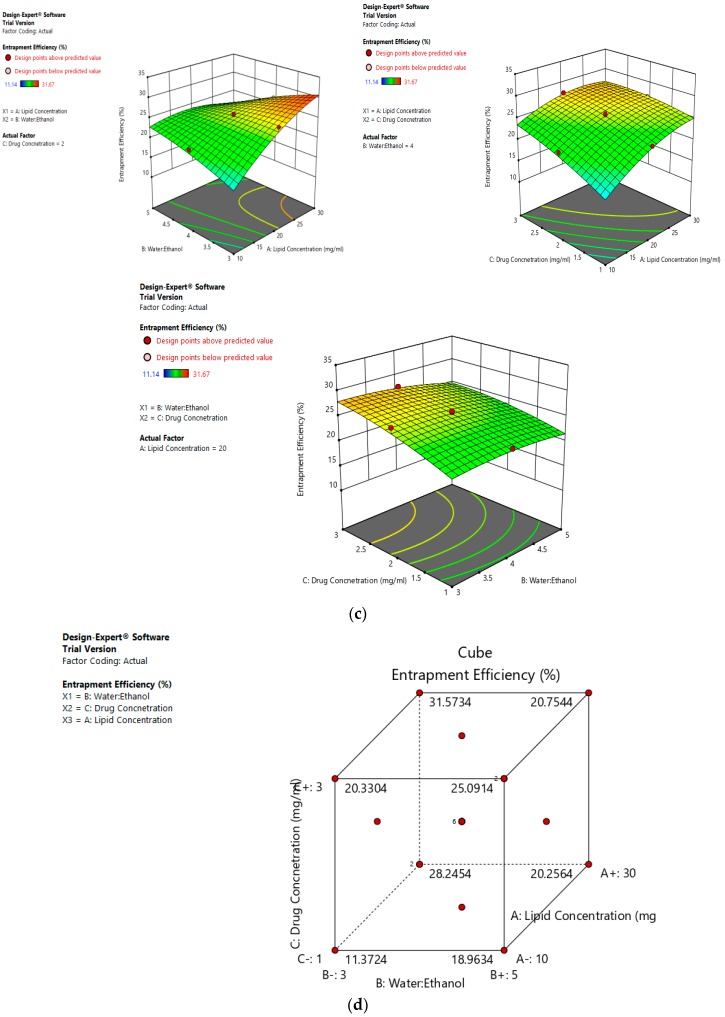
Effect of factors on entrapment efficiency of DL (**a**) Effect of individual factors on Entrapment Efficiency; (**b**) Effect of combinations of two factors on Entrapment Efficiency (2D plots); (**c**) Effect of combinations of two factors on Entrapment Efficiency (3D plots); (**d**) Effect of all three factors on Entrapment Efficiency.

On the other hand, a greater water:ethanol ratio leads to smaller liposomes, which results in lesser drug entrapment. Figure 3a–d illustrates the effect of factors on the entrapment efficiency of dacarbazine liposomes.

##### Risk Assessment (First Level)

**Figure 4:**
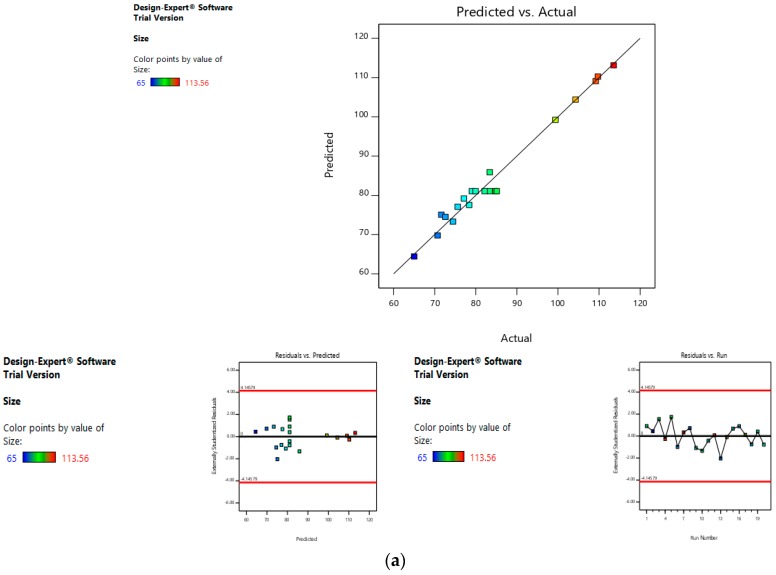

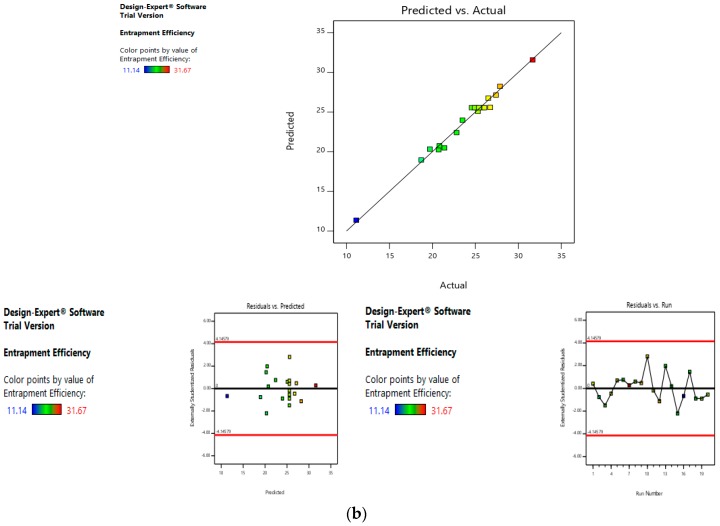
Risk assessment (first level) (**a**) with respect to size. (**b**) with respect to entrapment efficiency.

Risk assessment is employed to identify and establish the effect of materials and process variables on the final characteristics of the formulation. In the given case, the effect of lipid concentration, water:ethanol ratio and drug concentration was assessed on the size and entrapment efficiency of the liposomes. The linear curve of predicted versus actual response, and the symmetrical distribution pattern in residual versus predicted and residual versus run graph for particle size and entrapment efficiency of the drug, suggested that the model applied was fit, and the chances of missing other variables that might affect CQAs of the final liposomes were low (Figure 4a,b).

##### Selection of Optimum Formulation

An optimum formulation of dacarbazine-loaded liposomes was identified through numerical optimization by setting the constraints on dependent variables, size (minimum), and entrapment efficiency (maximum). The software suggested 41 solutions, out of which the one with the highest desirability factor was selected. The selected formulation suggested 13.168 mg/mL of lipid concentration, five as water:ethanol ratio, and 3 mg/mL of drug concentration.

The predicted size of the optimized formulation was 69.093 nm, and the obtained size was 74.66 nm; the predicted entrapment efficiency of the optimized formulation was 25.436%, while the obtained entrapment efficiency was 24.19%. Therefore, it can be seen that the predicted and obtained values are near.

#### 3.2.2. Second Level

For the second level, the optimum values of lipid concentration (13.168 mg/mL) and water:ethanol ratio (5) were already known from the results of the first level. Here, the two independent variables’ dacarbazine concentration and eugenol concentration were optimized. The dependent variables were the size and ratio of entrapped eugenol to entrapped dacarbazine. The size was constrained at a minimum. The r, the ratio of entrapped eugenol to entrapped dacarbazine, was constrained at maximum. It is because dacarbazine is a very potent anti-cancer drug, having a very low anti-cancer dose (2–4.5 mg/kg), while eugenol is an herbal agent with a high anti-cancer dose as compared to dacarbazine; researchers have introduced 100–125 mg/kg of eugenol in melanoma-bearing mice to observe anti-melanoma effects [34]. Therefore, since both the drugs are to be entrapped in the same formulation, the ratio of entrapped eugenol to entrapped dacarbazine should be as high as possible, so that maximum amount of eugenol is administered when the anti-cancer dose of dacarbazine is given to the animals (through liposomes).

When the minimum and maximum values of independent variables were put into the CCD statistical experimental design, the software suggested 13 runs with five center points. The formulations were made, analyzed, and the values of the dependent variables were put into the table (Table 6). Same as the first level, polynomial equations were generated to predict the effect of individual factors and combinations of factors on dependent variables.

##### Response Analysis for Optimization

On fitting the response values to various models, the data were best explained by the linear model (Table 2). Values of Model *F*-value, Lack of fit *F*-value and *p*-value were also favorable (Table 2).

Equation (7) represents the effect of independent variables on the size of the final liposomes.

Size = +140.34 + 12.76A + 26.99B(7)

**Figure 5:**
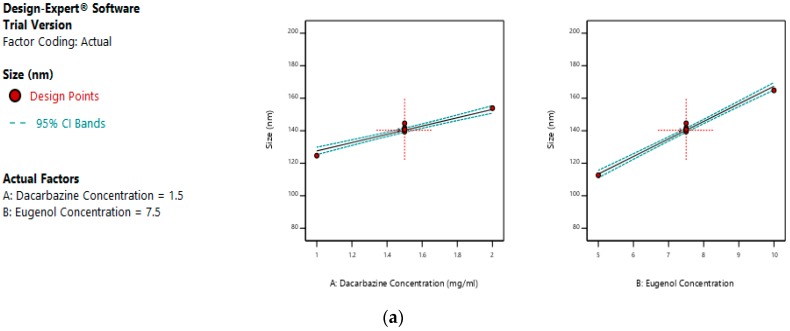

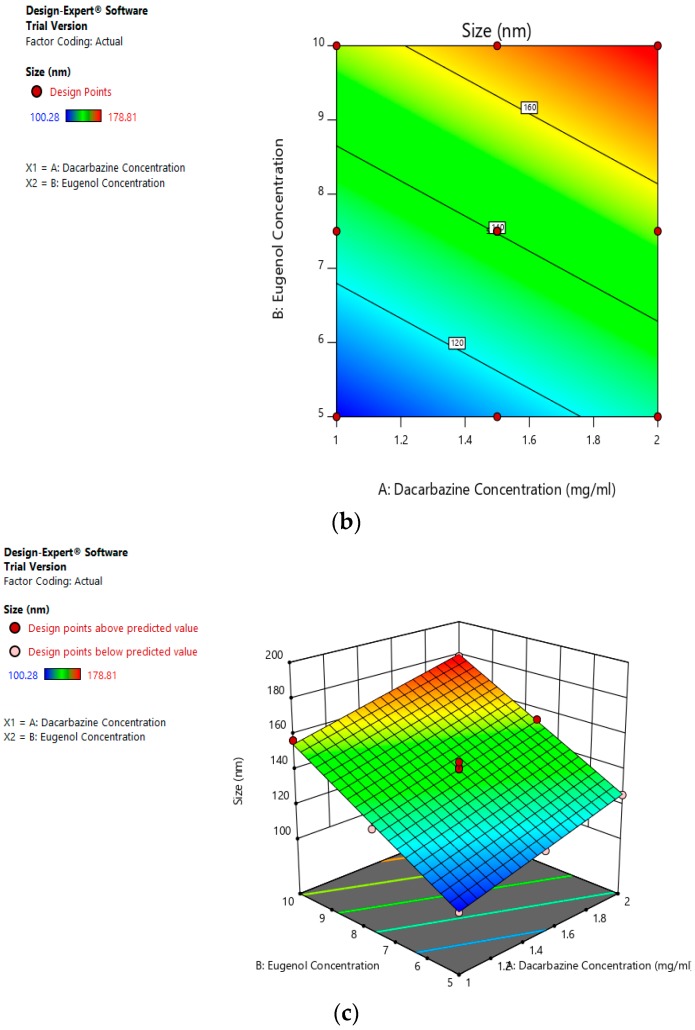
Effect of factors on size of DEL (**a**) Effect of individual factors on the size of dual loaded liposomes; (**b**) Effect of the combination of two factors on the size of dual loaded liposomes (2D plot); (**c**) Effect of the combination of two factors on the size of dual loaded liposomes (3D plot).

The mean size ranged from 100.28 nm to 178.81 nm. The equation suggests that the size of the liposomes is more influenced by eugenol concentration. The linear terms of dacarbazine concentration and eugenol concentration both have a positive effect on the size of the liposomes. Thus, the size of the liposomes increases with an increase in any of the drug concentration. Figure 5a–c represents the effect of independent variables on the size of dual drugs-loaded liposomes.

Eugenol:Dacarbazine was constrained at maximum. On fitting the response values to various models, the data was best explained with the quadratic model (Table 2). Values of Model *F*-value, Lack of fit *F*-value and *p*-value were also favorable (Table 2).

Equation (8) represents the effect of independent variables on the size of the final liposomes.

Eugenol:Dacarbazine = +2.29 − 0.5733A + 0.2883 B − 0.1200 AB + 0.1210 A^2^ − 0.1440 B^2^ (8)

**Figure 6:**
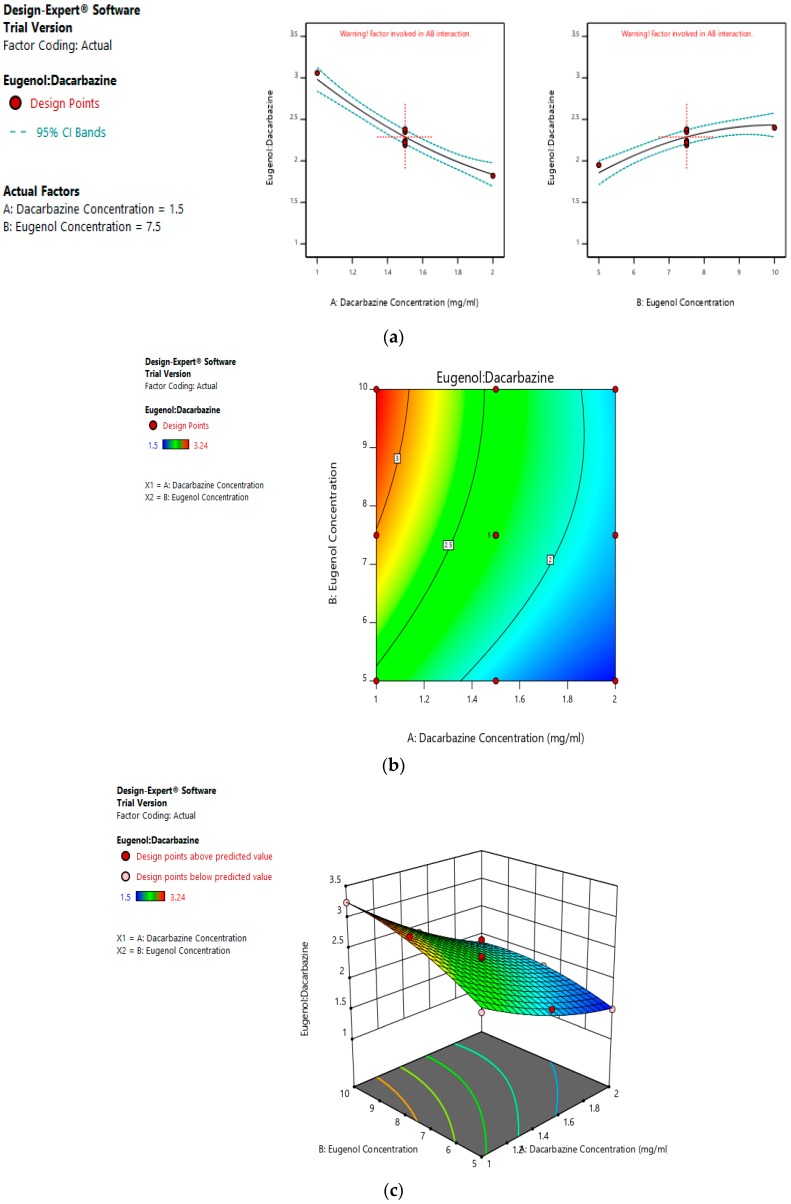
Effect of factors on Eugenol:Dacarbazine ratio of DEL (**a**) Effect of individual factors on Eugenol: Dacarbazine ratio of dual loaded liposomes; (**b**) Effect of combination of two factors on Eugenol: Dacarbazine ratio of dual loaded liposomes (2D plot); (**c**) Effect of Effect of combination of two factors on Eugenol:Dacarbazine ratio of dual loaded liposomes (3D plot).

Values of the ratio of entrapped eugenol to entrapped dacarbazine range between 1.5 and 3.24. As suggested by the equation, the linear term of dacarbazine concentration has a negative effect, and the linear term of eugenol concentration has a positive effect on entrapped eugenol:dacarbazine; while dacarbazine concentration has a more pronounced effect. Figure 6a–c represents the effect of independent variables on the entrapped Eugenol:Dacarbazine ratio of dual drugs-loaded liposomes.

##### Risk Assessment (Second Level)

**Figure 7:**
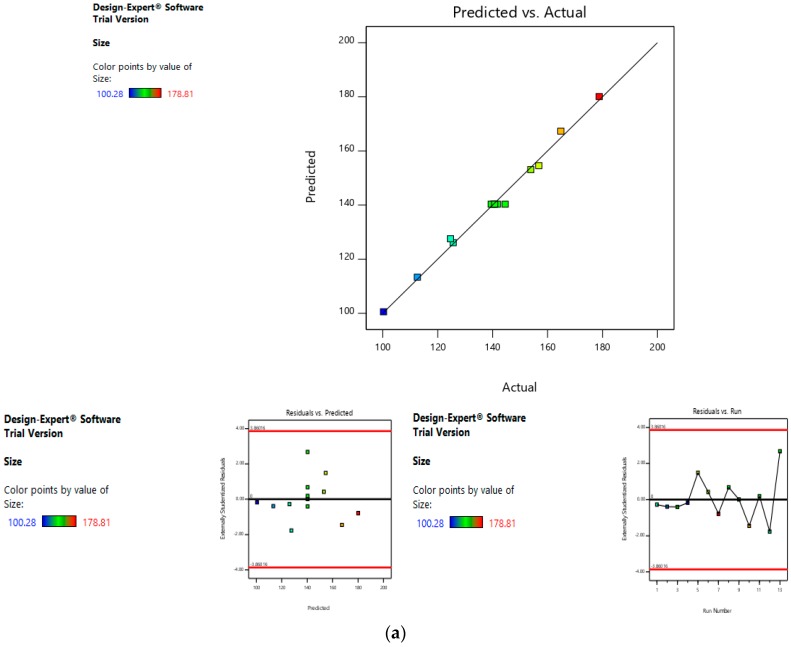

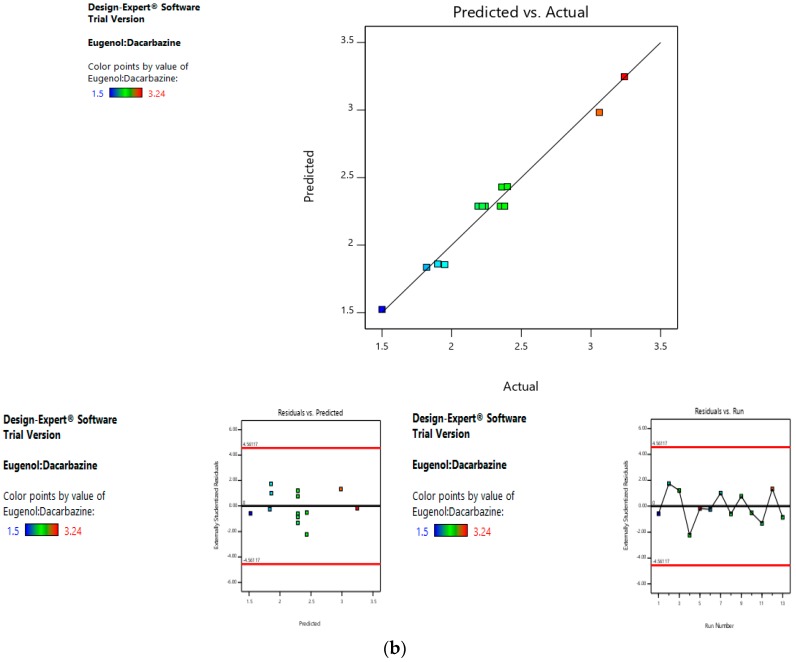
Risk assessment (second level) (**a**) with respect to size. (**b**) with respect to Eugenol:Dacarbazine.

In the second level of QbD, the effect of dacarbazine concentration and eugenol concentration was assessed on the size and entrapment efficiency of the liposomes. The linear curve of predicted versus actual response, and symmetrical distribution pattern in residual versus predicted, and residual versus run graph for particle size and eugenol:dacarbazine, suggested that the model applied was fit, and the chances of missing other variables that might affect CQAs of the final liposomes were low (Figure 7a,b).

##### Selection of Optimum Formulation

Optimum formulation of dual-loaded liposomes was identified through a numerical optimization by setting the constraints on dependent variables, size (minimum), and eugenol:dacarbazine (maximum). The software suggested 15 solutions, out of which the one with the highest desirability factor was selected. The selected formulation suggested 1 mg/mL of dacarbazine concentration, and 6.882 mg/mL of eugenol concentration. The predicted size of the DEL synthesized by the suggested formula was 120.889 nm, and the size obtained was 124.0 nm. The predicted eugenol:dacarbazine value was 2.873, while it was practically observed to be 2.906. So, the predicted and obtained values were found to be in agreement.

### 3.3. Surface Functionalization of Optimized Formulation

Different concentrations of HA solution were used to coat the optimized cationic liposomes, and the size of the final coated liposomes was assessed in each case to determine the optimum concentration of HA solution to be used. With 0.005% solution, only a negligible increase in size was observed. When 0.01% HA solution was used, the size of the liposomes increased from 124.0 nm to 159.5 nm. With 0.05% and 0.1% HA solutions, the size increased to 235.7 nm and 364.1 nm, respectively. Since the increase in size was too large, these two concentrations were discarded. Increase in size with 0.005% HA solution was too less to indicate any significant coating. Hence, 0.01% concentration was selected as the optimum concentration of HA solution. The liposomes coated with 0.01% HA solution were later analyzed by TEM and SEM to visualize and confirm a significant coating of HA.

### 3.4. Particle Size, Size Distribution

Blank liposomes had a size of 54.00 ± 1.73 nm, which increased to 74.66 ± 2.71 nm when dacarbazine was loaded. When both the drugs were loaded, the size further increased to 124.00 ± 4.26 nm. After surface functionalization of the optimized dual loaded liposomes, the size remarkably increased to 159.5 ± 3.62 nm. This is the reason that every effort was made from the beginning to achieve the minimum size at every step. PDI, which is a measure of the size distribution of the nanoparticles, was within a suitable range for all the formulations.

### 3.5. Zeta Potential

As the zeta potential is one of the major determinants of the stability of the nanoformulations, it too was determined. Zeta potential of BL was found to be −9.97 mV, which indicates a moderate stability of the liposomes. The zeta potential of DEL was found to be −8.70, which is nearly the same as that of the blank liposomes. Since both the drugs are entrapped inside the liposomes, the surface charge remains more or less unaltered.

After coating of liposomes with HA (DELC), the zeta potential was −12.8. Here, the negative charge is supposed to be due to the carboxylic groups of HA present on the surface of the liposomes. This increase in the negative charge of the liposomes indicates towards a successful coating of the liposomes with the electronegative HA.

### 3.6. Electron Microscopy

**Figure 8:**
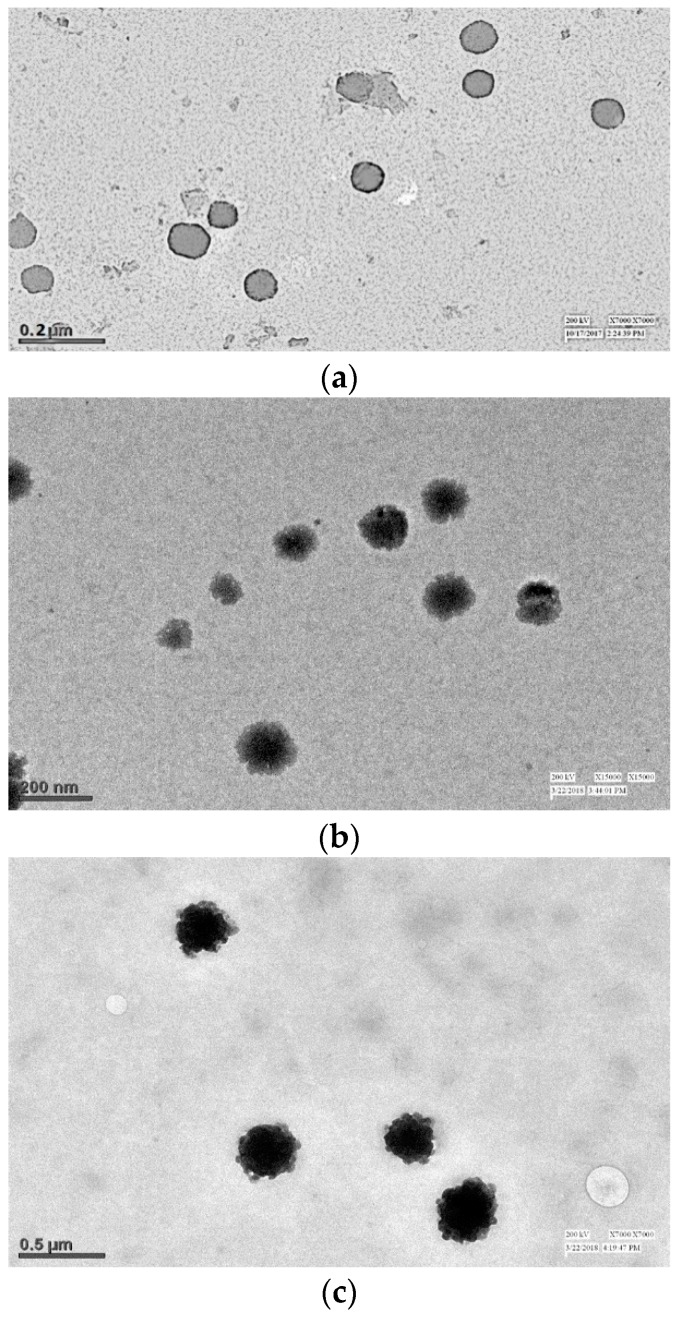
Transmission Electron Microscopy (TEM) images of (**a**) Blank Liposomes (**b**) Dacarbazine and Eugenol Liposomes (**c**) Dual loaded surface-functionalized liposomes. (**a**) Liposomes appear hollow with thin lipid bimembrane (**b**) Liposomes have darker core and thickened lipid bimembrane indicating towards loading of both drugs (**c**) Liposomes have irregular surface indicating surface coating.

Figure 8 shows the TEM images of (a) BL, (b) DEL, and (c) DELC. As it can be seen in the images, the blank liposomes are hollow structures bounded by a thin lipid bilayer. However, the DEL have drug entrapped in the core, as well as in the lipid bilayer, which is evident by the thickening of the bilayer. In the image of the DELC, the surface of the liposomes is irregular which confirms the coating of liposomes by HA.

**Figure 9:**
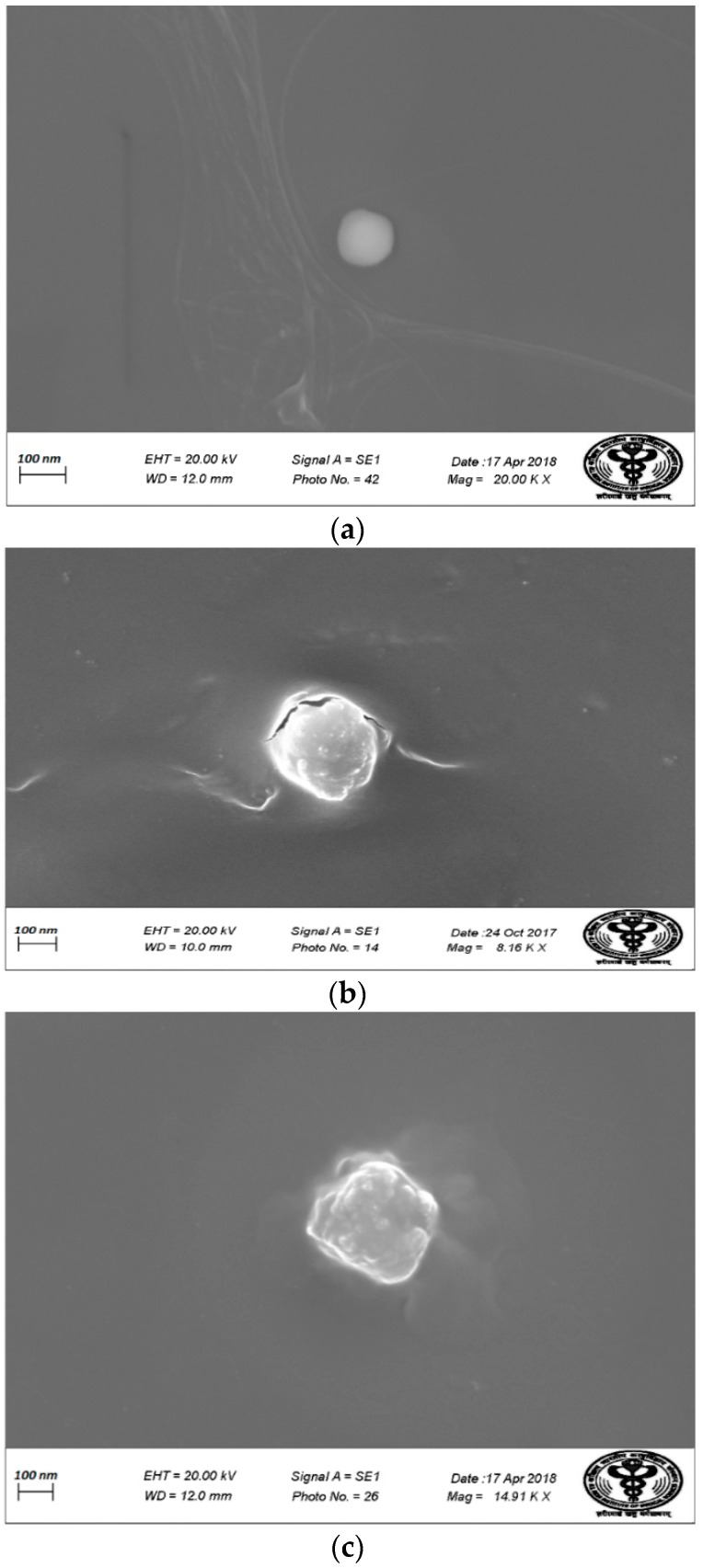
Scanning Electron Microscopy (SEM) images of (**a**) Blank Liposomes; (**b**) Dacarbazine and Eugenol Liposomes; (**c**) Dual loaded surface-functionalized liposomes. (**a**) More spherical and round liposomes (**b**) Liposomes are bigger in size (**c**) Surface of liposomes appear irregular due to surface coating.

Figure 9 shows the SEM images of (a) BL, (b) DEL, and (c) DELC. SEM images are also in agreement with the TEM images, showing a somewhat smaller and regular structure of blank liposomes, bigger structures of DEL, and bigger with the irregular surface of DELC. The sizes revealed by TEM and SEM analysis are also in agreement with the results of particle size analysis by zeta sizer. Also, SEM and TEM images revealed no aggregation of the liposomal structures.

### 3.7. Drug Loading

The absorbance of the ethanolic solution of drugs-loaded liposomes was measured at λ_1_^et^ and λ_2_^et^, and substituted into Equations (1) and (2) which were then solved to find out the values of C_d_^et^ and C_e_^et^. After multiplying these values with the dilution factor, the concentration of dacarbazine was found to be 4.374 mg, and the concentration of eugenol was found to be 12.714 mg in the liposomes. Calculating the percentages, the Dacarbazine loading was found to be 15.272%, and Eugenol loading was found to be 44.392%.

### 3.8. Entrapment Efficiency

Since the amount of dacarbazine initially added was 25 mg (1 mg/mL in 25 mL water), and the amount entrapped was 4.374 mg, the entrapment efficiency of dacarbazine was 17.49%.

The amount of Eugenol initially added was 34.41 mg (6.882 mg/mL in 5 mL ethanol), and the amount entrapped was 12.714 mg, so the entrapment efficiency of eugenol was 36.94%.

### 3.9. Drug Release

**Figure 10:**
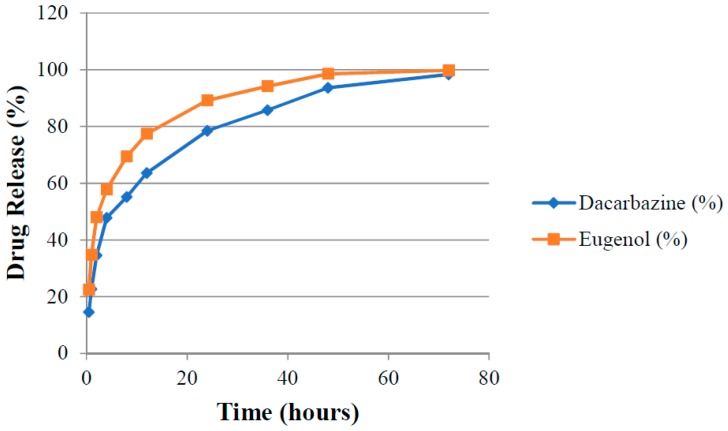
Cumulative release of Dacarbazine and Eugenol from uncoated liposomes.

**Figure 11:**
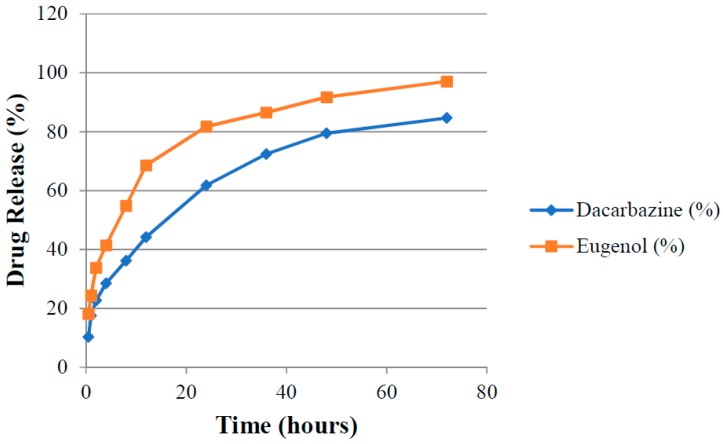
Cumulative release of Dacarbazine and Eugenol from hyaluronic acid (HA)-coated liposomes.

Absorbance (A_1_ and A_2_) of aliquots of release media withdrawn at different time points were measured at λ_1_ and λ_2_ against pure release media as blank. The amount of drugs present (cumulative amount of drugs released) in the release media at different time intervals was calculated by putting these absorbance values in Equations (3) and (4). Release rates of drugs were determined for both uncoated (DEL) and HA-coated (DELC) liposomes. Plots were made between the time interval and cumulative percent of drug released from DEL (Figure 10) and DELC (Figure 11). As can be seen from the plots, the release of drugs was more sustained from DELC, indicating the role of HA-coating in slowing down the release of drugs. From DEL, in 12 h, 63.6 ± 2.04% of dacarbazine and 77.36 ± 2.74% of eugenol was released, while from DELC, only 44.24 ± 2.55% of dacarbazine and 68.62 ± 3.20% of eugenol was released in 12 h. In 24 h, the cumulative released amount was found to be 78.46 ± 4.7% of dacarbazine and 89.21 ± 3.64% of eugenol from DEL, and 61.78 ± 3.74% of dacarbazine and 81.73 ± 2.10% of eugenol from DELC. 98.36 ± 1.21% dacarbazine and 99.73 ± 0.18% eugenol were released in a span of 72 h from DEL. 84.67 ± 2.64% dacarbazine and 97.1 ± 1.78% eugenol were released from DELC in 72 h. Thus, the release of both of the drugs from DEL and DELC was found to be sustained, but DELC showed more sustained drugs release, owing to HA-coating on the surface.

### 3.10. Stability Study

The size, PDI and drug content of lyophilized liposomal formulation were studied for four weeks, and results are summarized in Table 7. Liposomes were found to be fairly stable as they did not show any remarkable increase in size or PDI; neither had they shown a significant reduction in their drug content. This implies that, in the lyophilized form and under suitable storage conditions, formulated liposomes were able to retain their size without any leakage or leeching of drugs.

To check the stability of liposomes in cell culture media (DMEM + 10% FBS), lyophilized liposomes were also dispersed in it, and the above mentioned parameters were measured for three days. Liposomes retained their stability in cell culture media also (Table 8).

### 3.11. Cell Line Studies

#### 3.11.1. MTT Assay

**Figure 12:**
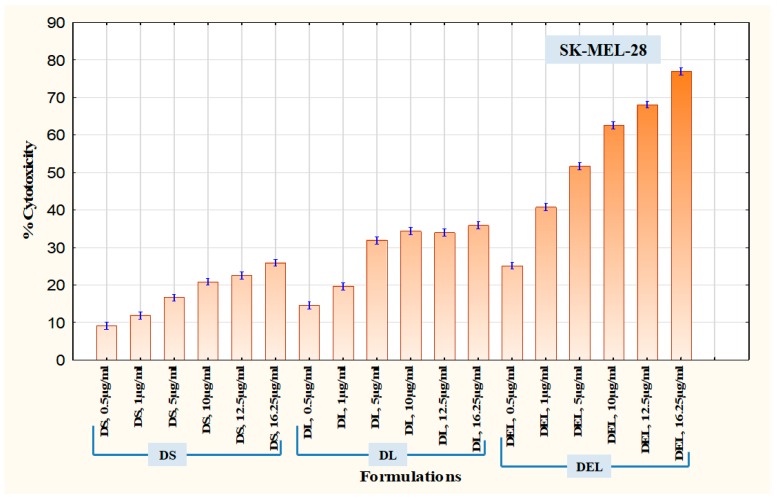
Cytotoxicity in SK-MEL-28 Melanoma Cells.

An MTT assay was performed to assess and compare the cytotoxic potential of the formulations. This test is based on the quantification of formazan dye which is produced after the metabolic cleavage of yellow tetrazolium salt MTT (3-(4,5-dimethylthiazol-2-yl)-2,5-diphenyl tetrazolium bromide assay) by alive cells [31]. First, the cytotoxicity of DEL was compared with DS and DL in SK-MEL-28 cells. As it was known that eugenol inhibits survivin protein, thereby inducing apoptosis and inhibiting angiogenesis, SK-MEL-28 cells were chosen because they overexpress survivin protein [35]. The amounts of formulations were introduced according to the amount of dacarbazine they contained. The different concentrations of dacarbazine (in each formulation) tested on SK-MEL-28 cells were 0.5 µg/mL, 1 µg/mL, 5 µg/mL, 10 µg/mL, 12.5 µg/mL, and 16.25 µg/mL. Results of MTT assay in SK-MEL-28 cells are graphically represented in Figure 12. DS produced cytotoxicity of only 25.91 ± 1.060% at a concentration of 16.25 µg/mL, while DL at the same concentration of dacarbazine produced cytotoxicity of 35.89 ± 1.109%. In contrast to these, DEL produced cytotoxicity of 76.96 ± 0.351% (at the same concentration of dacarbazine), which was more than double the cytotoxicity of DL. This suggests that eugenol is quite capable of potentiating the effect of dacarbazine on melanoma cells. This also indicates that combining eugenol with dacarbazine may allow a significant reduction of dacarbazine dose during chemotherapy. Since dacarbazine is a cytotoxic drug, reduction in its dose implies a reduction in unwanted toxicity on normal body cells.

**Figure 13:**
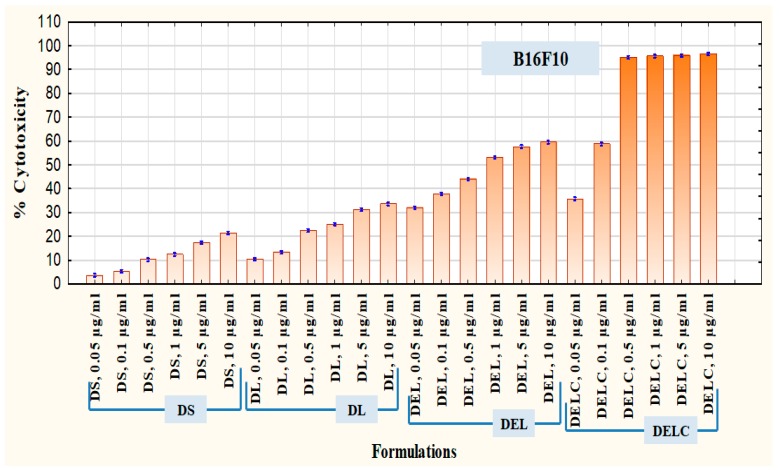
Cytotoxicity in B16F10 Melanoma Cells.

After SK-MEL-28 cells, B16F10 cells were employed to assess the therapeutic superiority of final formulation Dacarbazine + Eugenol Liposomes (DELC) over DS, DL, and DEL. B16F10 melanoma cells are also known to overexpress survivin and CD44 receptors [11,36]. Here the concentrations (of dacarbazine) tested were 0.05 µg/mL, 0.1 µg/mL, 0.5 µg/mL, 1 µg/mL, 5 µg/mL, and 10 µg/mL. Lower concentrations were tested this time because more potent action was expected from DELC. The results are presented in Figure 13. As can be observed, at a concentration of 0.05 µg/mL, DS produced cytotoxicity of 3.60 ± 0.055%, while DELC produced cytotoxicity of 35.72 ± 0.466%. At a concentration of 0.5 µg/mL, cytotoxicity of DS was only 10.20 ± 0.288%, whereas DELC produced 95.08 ± 0.310% cytotoxicity. Therefore, our final coated liposomes of Dacarbazine and Eugenol showed enhancement of more than 900% in cytotoxicity, as compared to that of Dacarbazine Solution at the same concentration of dacarbazine (0.5 µg/mL) in both the formulations. Therefore, this concept of combining dacarbazine with eugenol, and encapsulating both the drugs in surface-functionalized liposomes (for better targeting and increased uptake), is capable of enabling oncologists to decrease the dose of dacarbazine several times. This can be a breakthrough in the chemotherapy of melanoma, as this will lead to better therapeutic outcomes with a much lesser dose of the cytotoxic agent and hence much lesser side effects.

Cytotoxicity of blank liposomes was also assessed in both the cell lines to ascertain the safety of the carrier system (data not shown). The blank liposomes were found to be safe with no significant cytotoxicity.

#### 3.11.2. Apoptosis Assay

Apoptosis caused by the formulations can be measured through a Phosphatidylserine (PS) assay. PS is normally located on the cytoplasmic face of the plasma membrane. During apoptosis, PS translocates to the outer leaflet of the plasma membrane, and can be detected by flow cytometry and cell imaging through binding to fluorochrome-labeled Annexin V when calcium is present [37]. An apoptosis assay was performed on SK-MEL-28 cells using an Annexin V kit. The concentration of dacarbazine in all the formulations was ten µg/mL. In DS treated cells, the percentage of viable, early apoptotic, late apoptotic and necrotic cells were 78.2 ± 0.1, 6.66 ± 0.115%, 8.43 ± 0.057% and 6.66 ± 0.057% respectively. In the cells treated with DL, the percentage of early apoptotic, late apoptotic and necrotic cells were 17.2 ± 0.1%, 23.2 ± 0.1% and 5.3 ± 0.2% respectively, while viable cells were 54.53 ± 0.057%. The viability of cells decreased to 38.3 ± 0.1% when treated with DEL. Early apoptotic, late apoptotic and necrotic cells in the DEL-treated group were at 6.36 ± 0.057%, 39.23 ± 0.057% and 16.1 ± 0%. While the performance of DLC was slightly inferior to DEL, it performed much better than DL. 

**Figure 14:**
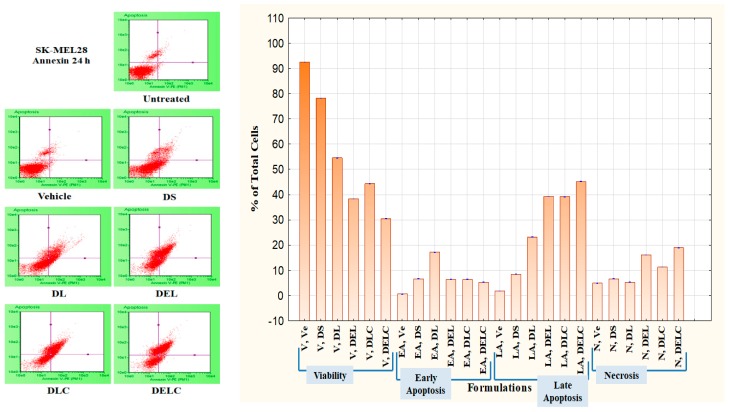
Apoptosis in SK-MEL-28 Melanoma cells.

**Figure 15:**
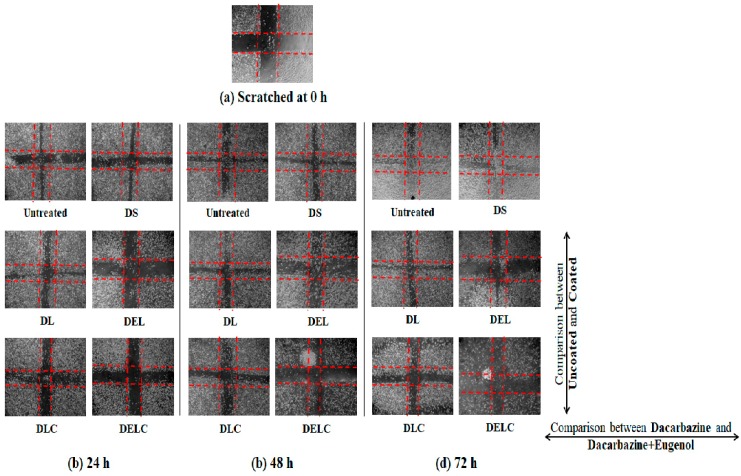
Migration in EA.hy926 cells.

This can be owed to better uptake of liposomes by melanoma cells due to HA coating, and thus indicates towards the importance of surface functionalization. Furthermore, DELC caused maximum late apoptosis (45.16 ± 0.057%) and necrosis (19.0 ± 0.1%) out of all the formulations because of a better uptake of the liposomes which contained both dacarbazine and eugenol. So what can be concluded here is that the number of early apoptotic cells decreased (in addition to viability), while there was a significant increase in the number of late apoptotic and necrotic cells after the inclusion of Eugenol into the liposomes. Shibuya et al. had reported that Dacarbazine induced only apoptosis and no necrosis up to the concentration of 40 µg/mL [38]. In our study, also, Dacarbazine did not induce necrosis, as is evident by the fact that there is no significant difference in the number of necrotic cells in untreated, and DS- and DL-treated groups. However, there was a significant increase in the fraction of necrotic cells after coating of the liposomes, which implies that coating resulted in a higher concentration of dacarbazine reaching the cells. The increase was even higher after the inclusion of Eugenol. Results are represented in Figure 14 and Figure 15.

Dacarbazine, which is an alkylating chemotherapeutic agent, mainly kills cancer cells by inducing apoptosis and necrosis [39]. However, its effects are not observed to a significant level of its potential, because of the anti-apoptotic ability of the cancer cells, which is imparted to them by survivin. Here, eugenol downregulated the survivin to inhibit the anti-apoptotic ability of melanoma cells. In the absence of survivin, dacarbazine released from the liposomes could exert its apoptotic and necrotic effects more efficiently.

#### 3.11.3. Migration Assay

**Figure 16:**
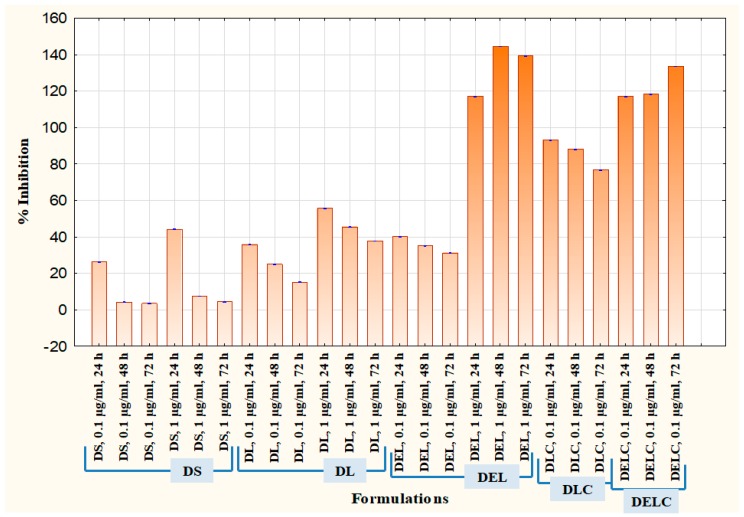
Migration assay in EA.hy926 cells.

Cell migration plays an important part in the invasion and metastasis of tumor to distant sites. Metastasis is a major complication of cancer, and is particularly more common in case of melanoma [40,41]. Melanoma, once metastasized, is almost always fatal [42]. Vascular endothelial cells play significant roles in many physiologically and pathologically important processes, and thus are commonly used to describe mechanisms of inflammation, angiogenesis, tumor growth, migration, and metastasis. EA.hy926 cell line, which is derived as the hybrid of primary human umbilical vein cells (HUVECs) and the continuous human lung carcinoma cell line A549, is presently the best characterized macro-vascular endothelial cell line [43]. Figure 15 and Figure 16 present the results of the migration study performed on EA.hy926 cells. The values of ‘% inhibition of migration’ are calculated concerning untreated, i.e., the migration of untreated cells is assumed to be 100% (inhibition is 0%), and all other values are relative to the migration of the untreated cells. The concentrations of DS, DL and DEL tested were 0.1 µg/mL and 1 µg/mL, while concentrations of DLC and DELC tested were 0.01 µg/mL (data not shown), and 0.1 µg/mL.

At one µg/mL, DS showed an inhibition of 44.13 ± 0.152% at 24 h, which was quite significant, but inhibition drastically decreased to only 7.43 ± 0.057% and 4.3 ± 0.1% at 48 h and 72 h respectively. In the case of DL (1 µg/mL), inhibition was 55.66 ± 0.115%, 45.43 ± 0.152% and 37.73 ± 0.057% at 24 h, 48 h and 72 h, respectively. This indicates towards the somewhat sustained release of dacarbazine from DL, as the decrease in performance was not as steep as DS. In the DEL (1 µg/mL) treated group, there was a remarkable migration inhibition of 116.96 ± 0.057%, 144.43 ± 0.115% and 139.13 ± 0.152% at three different time points. Here comes the role of eugenol, which has contributed significantly in inhibiting the migrating ability of the cells. As we can see, the % inhibition has increased from 24 h to 48 h, and then only slightly decreased at 72 h, this indicates towards the more sustained release of the drugs. In the DLC (0.1 µg/mL) treated group, inhibition was 93.13 ± 0.115%, 88.03 ± 0.57% and 76.7 ± 0.1%. Here, the action was sustained for 72 h, but not as much as in the case of DEL (inhibition has decreased rather than increasing). This shows that Eugenol plays a more significant role (than HA coating) in sustaining the release of dacarbazine. Inhibition in the cells treated with our final formulation DELC (0.1 µg/mL) was the highest; 116.86 ± 0.057%, 118.26 ± 0.115%, and 133.53 ± 0.0115%. 

It should be noted here that DELC gave similar results as DEL at a concentration one-tenth of it. This indicates that surface coating has improved the performance of the formulation by around ten times. Also, the inhibition continuously increased for 72 h, which implies most sustained action out of all the formulations. It is because of the presence of both; the eugenol and the HA coating.

One interesting point to be noted here is that DLC has performed better than DEL (comparing results of 0.1 µg/mL). This unexpected behavior can be attributed to the fact that EA.hy926 cells overexpress CD44 on the cell surface [44], and HA reportedly binds to EA.hy926 cells [45]. This must have resulted in better uptake of DLC as compared to DEL, which does not have HA coating to bind to CD44 receptors.

#### 3.11.4. Proliferation Assay

**Figure 17:**
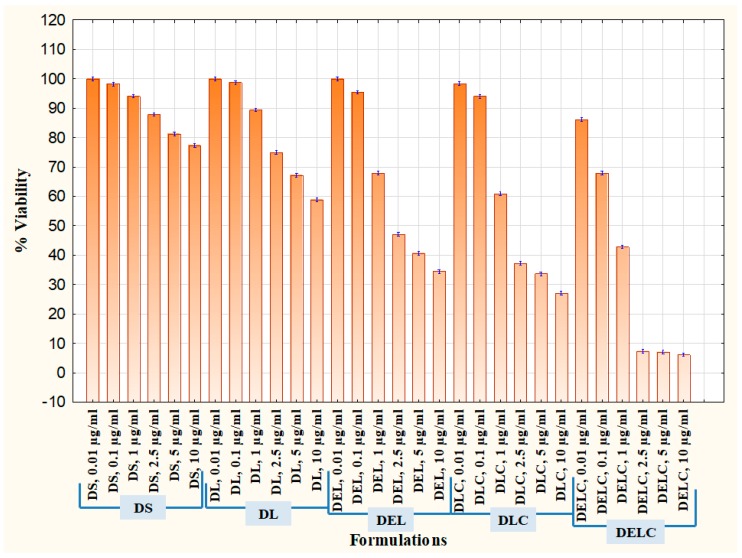
Proliferation Assay in EA.hy926 cells.

Cell proliferation is the rapid increase in the number and amount of cells. Cytotoxic anti-cancer drugs kill cells that have a high basal level of proliferation and regeneration [46]. Thus, it is important to assess the effect of eugenol in the enhancement of proliferation when added to dacarbazine. It is usually more effective to test the antiangiogenic potential of pharmaceutical formulations on cells which have a substantial rate of proliferation [47], and so EA.hy926 cells were employed for the proliferation assay also. The results are graphically represented in Figure 17, where % viability of cells after treatment with different formulations is denoted. Concentrations tested were 0.01 µg/mL, 0.1 µg/mL, 0.5 µg/mL, 1 µg/mL, 2.5 µg/mL, 5 µg/mL, and 10 µg/mL. DS at dacarbazine concentration of 10 µg/mL reduced the viability of cells to 77.31 ± 0.47, while DL reduced it to 58.83 ± 0.610%. When eugenol was co-loaded with dacarbazine in the liposomes (DEL), the cell viability decreased to 34.46 ± 0.643% at ten µg/mL. The viability of cells treated with DLC (10 µg/mL) was 27.08 ± 0.605%, which was a little less than DEL because of the same reason as stated above. Most interestingly, the coated liposomes of Dacarbazine and Eugenol (DELC) left only 6.14 ± 0.618% cells viable. The anti-proliferative performance of DELC was much more significant than DS, which is the conventional way of how the dacarbazine is administered.

## 4. Conclusions

Keeping the highly resistant and aggressive nature of melanoma in mind, dacarbazine- and eugenol-loaded liposomes were successfully developed for a combinatorial approach against melanoma. The QbD approach enabled us to synthesize the said anti-melanoma formulation with optimum parameters in the most logical manner. Applying this QbD approach at two levels further made the process easier to reproduce, and more cost-effective. Surface functionalization of the formulation made the entire therapy more targeted to spare normal body cells from unwanted toxicity. In-vitro characterization of the nanoliposomes ascertained the utility of the QbD application. This work is a good example and illustration of the successful application and value of the quality by design approach in the development of effective pharmaceutical nanoformulations.

The performance of the formulation as an anti-melanoma agent was assessed by cell line studies. Combining eugenol with dacarbazine resulted in much higher anti-melanoma activity of the formulation. This enhancement is supposed to be due to the inhibition of the anti-apoptotic protein surviving, which is overexpressed in the melanoma cells, and makes them resistant towards apoptosis. Including Eugenol resulted in a downregulation of survivin protein, consequent to which, dacarbazine could perform its function to its maximum potential. This resulted in significantly higher cytotoxicity, increased apoptosis, and much decreased migration and proliferation of the cancer cells. Thus, the combination of dacarbazine and eugenol holds the promise of overcoming the resistance of melanoma cells and any challenges of anti-melanoma therapies. In addition to increased apoptosis and cytotoxicity, this combination also promises to inhibit the metastatic potential of the melanoma.

Since this study has strongly indicated that survivin is inhibited in the presence of eugenol, the result of which is that dacarbazine could perform better, this opens up the scope of analyzing the survivin expression of the melanoma cells before and after treatment. Also, the efficacy of this novel approach can be tested in the in-vivo model to ascertain its applicability. Therefore, survivin expression studies and in-vivo studies can be future prospects to support the importance of the above-mentioned results and inferences.

## Figures and Tables

**Table 1:** Initial Risk Assessment (First level).

Variables	Relative Impact on CQAs		Suitable Range
	Size	Entrapment Efficiency	
**CMAs**			
Lipid Concentration	High	Medium	10 mg/mL–30 mg/mL
Drug Concentration	Medium	High	1 mg–3 mg
Lipid: Cholesterol	Low	Medium	2
**CPPs**			
Water: Ethanol	High	Medium	3–5
Stirring speed	Medium	Low	1000 rpm
Stirring time	Low	Low	60 min

**Table 2:** Design of Experiment (DOE) Variables (First level).

Independent Variables	Levels		
	−1	0	+1
Factor 1: Lipid Concentration (mg/mL)	10	20	30
Factor 2: Water:Ethanol	3	4	5
Factor 3: Drug Concentration (mg/mL)	1	2	3
**Dependent Variables**	**Constraints**		
Response 1: Size (nm)	Minimum		
Response 2: Entrapment Efficiency (%)	Maximum		
**Fixed Variables**	**Fixed Values**		
Stirring Time	60 min		
Stirring Speed	1000 rpm		
Cholesterol: Lipid	1:2		

**Table 3:** DOE Variables (Second level).

Independent Variables	Levels		
	−1	0	+1
Factor 1: Dacarbazine Concentration (mg/mL)	1	2	3
Factor 2: Eugenol Concentration (mg/mL)	5	7.5	10
**Dependent Variables**	**Constraints**		
Response 1: Size (nm)	Minimum		
Response 2: Eugenol:Dacarbazine	Maximum		
**Fixed Variables**	**Fixed Values**		
Lipid Concentration	13.168 mg/mL		
Water: Ethanol	5		
Stirring Time	60 min		
Stirring Speed	1000 rpm		
Cholesterol:Lipid	1:2		

**Table 4:** Response Analysis Data (Level 1). Values of Response 1 and Response 2 for 20 different combinations of Factor 1, Factor 2 and Factor 3.

	Factor 1	Factor 2	Factor 3	Response 1	Response 2
Run	Lipid Concentration (mg/mL)	Water:Ethanol	Dacarbazine Concentration (mg/mL)	Size (nm)	Entrapment Efficiency (%)
1	20	4	2	83.4 ± 2.61	25.85 ± 0.82
2	10	5	1	65 ± 2.12	18.7 ± 0.24
3	20	4	2	84.75 ± 3.5	24.57 ± 0.6
4	30	4	2	109.72 ± 5.34	26.5 ± 0.95
5	20	4	2	85.1 ± 4.24	26.05 ± 0.5
6	20	4	1	72.6 ± 2.73	22.82 ± 0.23
7	30	3	3	113.56 ± 3.21	31.67 ± 1.24
8	10	5	3	70.7 ± 1.75	25.3 ± 1.03
9	20	4	3	77.12 ± 2.03	27.4 ± 0.78
10	20	3	2	83.4 ± 2.3	26.73 ± 1.1
11	20	4	2	79.94 ± 3.18	25.42 ± 0.9
12	30	3	1	109.2 ± 3.32	27.87 ± 0.92
13	10	4	2	71.62 ± 1.54	21.4 ± 0.35
14	30	5	3	104.28 ± 3.76	20.82 ± 0.42
15	10	3	3	78.4 ± 1.24	19.7 ± 0.3
16	10	3	1	74.44 ± 2.37	11.14 ± 1.0
17	30	5	1	99.38 ± 3.5	20.72 ± 1.12
18	20	5	2	75.6 ± 2.64	23.5 ± 1.45
19	20	4	2	82.15 ± 2.35	24.92 ± 1.2
20	20	4	2	79.04 ± 1.25	25.16 ± 0.96

**Table 5:** Analysis of Variance (ANOVA) Analysis.

	Lack of Fit Tests			Model Summary Statistics				
Response	Model *F*-Value	Lack of Fit *F*-Value	*p*-Value	SD	*R* ^2^	Adjusted *R*^2^	Predicted *R*^2^	Suggested Model
**Level 1**								
**R1: Size**	57.79	1.36	<0.0001	2.72	0.9811	0.9642	0.9380	Quadratic
**R2: Entrapment Efficiency**	70.71	2.48	<0.0001	0.7394	0.9845	0.9706	0.9023	Quadratic
**Level 2**								
**R1: Size**	611.37	1.23	<0.0001	2.09	0.9919	0.9903	0.9866	Linear
***R*2: Eugenol:Dacarbazine**	68.80	1.14	0.0489	0.0863	0.9803	0.9663	0.8992	Quadratic

**Table 6:** Response data analysis (Second level). Values of Response 1 and Response 2 for 13 different combinations of Factor 1 and Factor 2.

	Factor 1	Factor 2	Response 1	Response 2
Run	Dacarbazine Concentration (mg/mL)	Eugenol Concentration (mg/mL)	Size (nm)	Eugenol:Dacarbazine
1	3	5	125.64 ± 3.63	1.5 ± 0.17
2	2	5	112.6 ± 4.7	1.95 ± 0.13
3	2	7.5	139.48 ± 3.23	2.38 ± 0.2
4	1	5	100.28 ± 2.74	2.36 ± 0.17
5	1	10	156.82 ± 5.13	3.24 ± 0.21
6	3	7.5	153.9 ± 4.85	1.82 ± 0.11
7	3	10	178.81 ± 6.72	1.9 ± 0.08
8	2	7.5	141.74 ± 2.74	2.24 ± 0.18
9	2	7.5	140.35 ± 4.27	2.35 ± 0.15
10	2	10	164.83 ± 6.83	2.4 ± 0.14
11	2	7.5	140.73 ± 2.2	2.19 ± 0.14
12	1	7.5	124.66 ± 4.28	3.06 ± 0.24
13	2	7.5	144.57 ± 3.5	2.22 ± 0.15

**Table 7:** Storage Stability.

Stability Parameters		0 Weeks	1 Weeks	2 Weeks	3 Weeks	4 Weeks
Size (nm)		124.0 ± 3.62	126.47 ± 2.16	131.8 ± 4.26	138.13 ± 6.1	147.94 ± 6.73
poly dispersity index (PDI)		0.214 ± 0.062	0.238 ± 0.040	0.277 ± 0.086	0.304 ± 0.081	0.316 ± 0.094
Drug Content (%)	Dacarbazine	15.272 ± 0.75	15.218 ± 0.81	15.132 ± 1.03	14.95 ± 1.45	14.824 ± 1.65
	Eugenol	44.392 ± 1.4	43.93 ± 1.82	42.30 ± 3.04	41.046 ± 2.5	40.174 ± 3.67

**Table 8:** Stability in Cell Culture Media (Eagle’s minimal essential medium (DMEM) + 10% FBS).

Stability Parameters		0 Day	1 Day	2 Days	3 Days	4 Weeks
Size (nm)		127.4 ± 1.80	132.64 ± 4.69	129.28 ± 3.75	134.61 ± 2.57	133.24 ± 4.35
PDI		0.175 ± 0.068	0.256 ± 0.055	0.222 ± 0.072	0.274 ± 0.075	0.302 ± 0.890
Drug Content (%)	Dacarbazine	14.76 ± 0.36	14.48 ± 0.32	14.38 ± 0.87	14.15 ± 1.06	13.98 ± 0.94
	Eugenol	42.45 ± 2.50	42.83 ± 1.68	41.32 ± 2.11	41.43 ± 1.65	41.57. ± 0.79

## References

[1] Sadozai H., Gruber T., Hunger R.E., Schenk M. (2017). Recent Successes and Future Directions in Immunotherapy of Cutaneous Melanoma. Front. Immunol..

[2] Bandarchi B., Jabbari C.A., Vedadi A., Navab R. (2013). Molecular biology of normal melanocytes and melanoma cells. J. Clin. Pathol..

[3] Fitzmaurice C., Allen C., Barber R.M., Barregard L., Bhutta Z.A., Brenner H., Dicker D.J., Chimed-Orchir O., Dandona R., Dandona L. (2017). Global, regional, and national cancer incidence, mortality, years of life lost, years lived with disability, and disability-adjusted life-years for 32 cancer groups, 1990 to 2015: A systematic analysis for the global burden of disease study. JAMA Oncol..

[4] Bhatia S., Tykodi S.S., Thompson J.A. (2009). Treatment of Metastatic Melanoma: An Overview. Oncol. Williston Park N.

[5] Mishra H., Mishra P.K., Ekielski A., Jaggi M., Iqbal Z., Talegaonkar S. (2018). Melanoma treatment: From conventional to nanotechnology. J. Cancer Res. and Clin. Oncol..

[6] Mishra H., Mishra P.K., Ekielski A., Iqbal Z., Jaggi M., Talegaonkar S. (2018). Functionalized nanoliposomes loaded with anti survivin and anti angiogenic agents to enhance the activity of chemotherapy against melanoma by 4-pronged action. Med. Hypotheses.

[7] Kesharwani S.S., Kaur S., Tummala H., Sangamwar A.T. (2018). Overcoming multiple drug resistance in cancer using polymeric micelles. Expert Opin. Drug Deliv..

[8] Kesharwani S.S., Kaur S., Tummala H., Sangamwar A.T. (2019). Multifunctional approaches utilizing polymeric micelles to circumvent multidrug resistant tumors. Colloids Surf. B Biointerfaces.

[9] Grossman D., McNiff J.M., Li F., Altieri D.C. (1999). Expression and targeting of the apoptosis inhibitor, survivin, in human melanoma. J. Investig. Dermatol..

[10] Helmbach H., Rossmann E., Kern M.A., Schadendorf D. (2001). Drug-resistance in human melanoma. Int. J. Cancer.

[11] Fernández J.G., Rodríguez D.A., Valenzuela M., Calderon C., Urzúa U., Munroe D., Rosas C., Lemus D., Díaz N., Wright M.C. (2014). Survivin expression promotes VEGF-induced tumor angiogenesis via PI3K/Akt enhanced β-catenin/Tcf-Lef dependent transcription. Mol. Cancer.

[12] Yamanaka K., Nakahara T., Yamauchi T., Kita A., Takeuchi M., Kiyonaga F., Kaneko N., Sasamata M. (2011). Antitumor activity of YM155, a selective small-molecule survivin suppressant, alone and in combination with docetaxel in human malignant melanoma models. Clin. Cancer Res..

[13] Ma W.-H., Liu Y.-C., Xue M.-L., Zheng Z., Ge Y.-L. (2016). Downregulation of survivin expression exerts antitumoral effects on mouse breast cancer cells in vitro and in vivo. Oncol. Lett..

[14] Zhang M., Sun Y.-F., Luo S. (2012). Ani-survivin DNAzymes inhibit cell proliferation and migration in Breast Cancer Cell Line MCF-7. Asian Pac. J. Cancer Prev..

[15] Al-Sharif I., Remmal A., Aboussekhra A. (2013). Eugenol triggers apoptosis in breast cancer cells through E2F1/survivin down-regulation. BMC Cancer.

[16] Slameňová D., Horváthová E., Wsólová L., Šramková M., Navarová J. (2009). Investigation of anti-oxidative, cytotoxic, DNA-damaging and DNA-protective effects of plant volatiles eugenol and borneol in human-derived HepG2, Caco-2 and VH10 cell lines. Mutat. Res. Toxicol. Environ. Mutagen..

[17] Carrasco A.H., Espinoza C.L., Cardile V., Gallardo C., Cardona W., Lombardo L., Catalán M.K., Cuellar F.M., Russo A. (2008). Eugenol and its synthetic analogues inhibit cell growth of human cancer cells (Part I). J. Braz. Chem. Soc..

[18] Mitra A.K., Agrahari V., Mandal A., Cholkar K., Natarajan C., Shah S., Joseph M., Trinh H.M., Vaishya R., Yang X. (2015). NOVEL DELIVERY APPROACHES FOR CANCER THERAPEUTICS. J. Control. Release Off. J. Control. Release Soc..

[19] Wickens J.M., Alsaab H.O., Kesharwani P., Bhise K., Amin M.C.I.M., Tekade R.K., Gupta U., Iyer A.K. (2017). Recent advances in hyaluronic acid-decorated nanocarriers for targeted cancer therapy. Drug Discov. Today.

[20] Wang Y., Yang F., Zhang H.-X., Zi X.-Y., Pan X.-H., Chen F., Luo W.-D., Li J.-X., Zhu H.-Y., Hu Y.-P. (2013). Cuprous oxide nanoparticles inhibit the growth and metastasis of melanoma by targeting mitochondria. Cell Death Dis..

[21] Camerin M., Moreno M., Marín M.J., Schofield C.L., Chambrier I., Cook M.J., Coppellotti O., Jori G., Russell D.A. (2016). Delivery of a hydrophobic phthalocyanine photosensitizer using PEGylated gold nanoparticle conjugates for the in vivo photodynamic therapy of amelanotic melanoma. Photochem. Photobiol. Sci..

[22] Deng C., Zhang Q., Fu Y., Sun X., Gong T., Zhang Z. (2017). Coadministration of Oligomeric Hyaluronic Acid-Modified Liposomes with Tumor-Penetrating Peptide-iRGD Enhances the Antitumor Efficacy of Doxorubicin against Melanoma. ACS Appl. Mater. Interfaces.

[23] Yu L.X. (2008). Pharmaceutical Quality by Design: Product and Process Development, Understanding, and Control. Pharm. Res..

[24] Xu X., Khan M.A., Burgess D.J. (2011). A quality by design (QbD) case study on liposomes containing hydrophilic API: I. Formulation, processing design and risk assessment. Int. J. Pharm..

[25] Senbanjo L.T., Chellaiah M.A. (2017). CD44: A Multifunctional Cell Surface Adhesion Receptor Is a Regulator of Progression and Metastasis of Cancer Cells. Front. Cell Dev. Biol..

[26] Sebaaly C., Jraij A., Fessi H., Charcosset C., Greige-Gerges H. (2015). Preparation and characterization of clove essential oil-loaded liposomes. Food Chem..

[27] Kumar S., Ali J., Baboota S. (2016). Design Expert ^®^ supported optimization and predictive analysis of selegiline nanoemulsion via the olfactory region with enhanced behavioural performance in Parkinson’s disease. Nanotechnology.

[28] Negi L.M., Jaggi M., Joshi V., Ronodip K., Talegaonkar S. (2015). Hyaluronan coated liposomes as the intravenous platform for delivery of imatinib mesylate in MDR colon cancer. Int. J. Biol. Macromol..

[29] Muley P., Kumar S., El Kourati F., Kesharwani S.S., Tummala H. (2016). Hydrophobically modified inulin as an amphiphilic carbohydrate polymer for micellar delivery of paclitaxel for intravenous route. Int. J. Pharm..

[30] Kumar S., Kesharwani S.S., Mathur H., Tyagi M., Bhat G.J., Tummala H. (2016). Molecular complexation of curcumin with pH sensitive cationic copolymer enhances the aqueous solubility, stability and bioavailability of curcumin. Eur. J. Pharm. Sci..

[31] Sharma H., Kumar K., Choudhary C., Mishra P.K., Vaidya B. (2016). Development and characterization of metal oxide nanoparticles for the delivery of anticancer drug. Artif. Cells Nanomed. Biotechnol..

[32] Jaafar-Maalej C., Diab R., Andrieu V., Elaissari A., Fessi H. (2010). Ethanol injection method for hydrophilic and lipophilic drug-loaded liposome preparation. J. Liposome Res..

[33] Lasic D.D. (1988). The mechanism of vesicle formation. Biochem. J..

[34] Ghosh R., Nadiminty N., Fitzpatrick J.E., Alworth W.L., Slaga T.J., Kumar A.P. (2005). Eugenol causes melanoma growth suppression through inhibition of E2F1 transcriptional activity. J. Biol. Chem..

[35] Chen Y., Kramer D.L., Li F., Porter C.W. (2003). Loss of inhibitor of apoptosis proteins as a determinant of polyamine analog-induced apoptosis in human melanoma cells. Oncogene.

[36] Mummert M.E., Mummert D.I., Ellinger L., Takashima A. (2003). Functional Roles of Hyaluronan in B16-F10 Melanoma Growth and Experimental Metastasis in Mice1. Mol. Cancer Ther..

[37] Prieto V.G., Sadick N.S., Shea C.R. (2002). Androgenetic Alopecia: Analysis of Proliferation and Apoptosis. Arch. Dermatol..

[38] Shibuya H., Kato Y., Saito M., Isobe T., Tsuboi R., Koga M., Toyota H., Mizuguchi J. (2003). Induction of apoptosis and/or necrosis following exposure to antitumour agents in a melanoma cell line, probably through modulation of Bcl-2 family proteins. Melanoma Res..

[39] Sanada M., Hidaka M., Takagi Y., Takano T.Y., Nakatsu Y., Tsuzuki T., Sekiguchi M. (2007). Modes of actions of two types of anti-neoplastic drugs, dacarbazine and ACNU, to induce apoptosis. Carcinogenesis.

[40] Feng T., Yu H., Xia Q., Ma Y., Yin H., Shen Y., Liu X. (2017). Cross-talk mechanism between endothelial cells and hepatocellular carcinoma cells via growth factors and integrin pathway promotes tumor angiogenesis and cell migration. Oncotarget.

[41] McKenzie J.A., Liu T., Goodson A., Grossman D. (2010). Survivin enhances motility of melanoma cells by supporting Akt activation and α5 integrin upregulation. Cancer Res..

[42] Tas F. Metastatic Behavior in Melanoma: Timing, Pattern, Survival, and Influencing Factors. https://www.hindawi.com/journals/jo/2012/647684/.

[43] Expression of Integrins and Adhesive Properties of Human Endothelial Cell Line EA.hy 926. http://cgp.iiarjournals.org/content/2/5/265.abstract.

[44] El-Dakdouki M.H., El-Boubbou K., Kamat M., Huang R., Abela G.S., Kiupel M., Zhu D.C., Huang X. (2014). CD44 Targeting Magnetic Glyconanoparticles for Atherosclerotic Plaque Imaging. Pharm. Res..

[45] Alam C., Seed M., Freemantle C., Brown J., Perretti M., Carrier M., Divwedi A., West D., Gustafson S., Colville-Nash P. (2005). The inhibition of neutrophil-endothelial cell adhesion by hyaluronan independent of CD44. Inflammopharmacology.

[46] Feitelson M.A., Arzumanyan A., Kulathinal R.J., Blain S.W., Holcombe R.F., Mahajna J., Marino M., Martinez-Chantar M.L., Nawroth R., Sanchez-Garcia I. (2015). Sustained proliferation in cancer: Mechanisms and novel therapeutic targets. Semin. Cancer Biol..

[47] Adair T.H., Montani J.-P. (2010). Angiogenesis Assays.

